# *MUC16* overexpression induced by gene mutations promotes lung cancer cell growth and invasion

**DOI:** 10.18632/oncotarget.24203

**Published:** 2018-01-12

**Authors:** Madiha Kanwal, Xiao-Jie Ding, Xin Song, Guang-Biao Zhou, Yi Cao

**Affiliations:** ^1^ Laboratory of Molecular and Experimental Pathology, Kunming Institute of Zoology, Chinese Academy of Sciences, Kunming, China; ^2^ Kunming College of Life Sciences, University of Chinese Academy of Sciences, Kunming, China; ^3^ Department of Cancer Biotherapy Center, The Third Affiliated Hospital of Kunming Medical University (Tumor Hospital of Yunnan Province), Kunming, China; ^4^ State Key Laboratory of Biomembrane and Membrane Biotechnology, Institute of Zoology, Chinese Academy of Sciences, Beijing, China

**Keywords:** air pollution-related lung cancer, MUC16 (CA125), gene mutation, CRISPR/Cas9 gene editing, biomarker

## Abstract

Air pollution is one of the leading causes of lung cancer. Air pollution-related lung cancer is a deteriorating public health problem, particularly in developing countries. The *MUC16* gene is one of the most frequently mutated genes in air pollution-related lung cancer. In the present study, *MUC16* mRNA expression was increased in ∼50% of air pollution-related lung cancer samples obtained from patients residing in air-polluted regions (Xuanwei and Fuyuan, Yunnan, China), and *MUC16* mRNA levels were correlated with the degree of air pollution. Furthermore, sequencing of the captured *MUC16* gene identified 561 mutation sites within the *MUC16* gene in the air pollution-related lung cancer tissues. Interestingly, some mutations at specific sites and one region were associated with *MUC16* mRNA up-regulation. Therefore, we further investigated the impacts of gene mutation on *MUC16* expressions and cell behaviors in cultured cells by inducing certain mutations within the *MUC16* gene using CRISPER/Cas9 genome editing technology. Certain mutations within the *MUC16* gene induced *MUC16* overexpression at both the mRNA and the protein level in the cultured cells. Additionally, *MUC16* overexpression induced by gene mutations had functional effects on the behavior of lung cancer cells, including increasing their resistance to cisplatin, promoting their growth, and enhancing their migration and invasion capabilities. Based on the data, we suggest that MUC16 mutations potentially associated with air pollution may participate in the development and progression of air pollution-related lung cancer. In addition to ovarian cancer, MUC16 may be a candidate biomarker for lung cancer.

## INTRODUCTION

Lung cancer is a leading cause of cancer-related death worldwide. Despite continuous efforts and improvements in the diagnosis and treatment of lung cancer, the overall survival rate is still very low [1]. Additionally, in most patients, lung cancer is already at an advanced stage upon diagnosis, causing single therapy to be mostly ineffective. Improved diagnostics and therapeutics for lung cancer are urgently needed, and novel tumour biomarkers must be discovered. Lung cancer is divided into non-small cell lung cancer (NSCLC) and small cell lung cancer. NSCLC accounts for 80% of all lung cancer cases, including adenocarcinoma (AD), squamous cell carcinoma (SCC), and large-cell carcinoma [2]. Smoking and air pollution are the main causes of lung cancer, and industrial development escalates the levels of air pollution, particularly in developing countries. Air pollution-related lung cancer is a deteriorating public health problem in developing countries [3]. In China, the rates of lung cancer incidence and mortality have increased rapidly in the past three decades. The lung cancer incidence rates in Xuanwei and Fuyuan in the Yunnan Province of China are among the highest in the country, which is attributed to severe air pollution exposure, specifically to polycyclic aromatic hydrocarbons (PAHs), in these regions [4–6]. The lung cancer cases in these areas are very good models for studying air pollution-related lung cancer [7]. Our previous study determined that mutations in the *MUC16* gene were observed in 50% of lung cancer patients residing in Xuanwei and Fuyuan, and the *MUC16* gene is among the top frequently mutated genes, thus providing a clue that MUC16 may be associated with air pollution-related lung cancer [6].

MUC16, also named CA125, belongs to mucin family, and mucins are involved in protecting and lubricating epithelial surfaces that line the internal organs of the body. In addition to their normal physiological role in protecting epithelial cells, mucins have been shown to participate in various diseases, including cancer [8]. MUC16, a cell surface glycoprotein with a variable number of tandem repeat structures, was first identified in 1981 [9]. MUC16 is a trans-membrane mucin that was originally detected in epithelial cells and in the mucus layer of the respiratory and gastrointestinal tracts. MUC16, which is cleaved and shed into the bloodstream, is actively researched as a serum biomarker for a variety of tumor types [10]. Greater than 80% of ovarian cancer patients exhibit significantly high MUC16 expression, and CA125 (MUC16) is currently the only serum tumor biomarker routinely used for the clinical diagnosis and predictor of prognosis for ovarian cancer. Additionally, MUC16 is also considered to be a gold standard marker for monitoring ovarian cancer recurrence [11, 12]. Although MUC16 was initially believed to be a specific biomarker of ovarian cancer, MUC16-related studies have clarified that this marker can also be detected in the sera of patients that have other types of cancer, including pancreatic cancer, colorectal cancer, and gastric adenocarcinoma [13, 14].

However, few studies have been conducted to clarify which MUC16 functions boost the development and progression of lung cancer. Additionally, studies regarding the regulatory mechanisms driving abnormal *MUC16* gene expression in cancer cells are very limited. Gene mutation is one of main mechanisms underlying gene up-regulation (the gain-of-function) or down-regulation (the loss-of-function). In the present study, we first analyzed *MUC16* mRNA expression in lung cancer tissues from patients residing in air-polluted regions (Xuanwei and Fuyuan). We then investigated the impacts of *MUC16* gene mutation on *MUC16* expression and cell behavior in cultured lung cancer cells by inducing certain mutations within this gene using CRISPR/Cas9 genome editing technology. Our study demonstrated that MUC16 up-regulation induced by gene mutations may be involved in the development and progression of lung cancer and that MUC16 may be a potential marker for diagnosis, predicting prognosis, monitoring recurrence, and guiding the treatment of NSCLC.

## RESULTS

### *MUC16* mRNA levels in NSCLC tissues were related to air pollution levels

To study the relationship between *MUC16* expression and the characteristics of lung cancer patients, we examined the *MUC16* mRNA levels in the 84 NSCLC tissues and their adjacent nonmalignant tissues obtained from patients residing in air-polluted regions (Xuanwei and Fuyuan) using qRT-PCR. Compared with those of their matched adjacent noncancerous tissues, the *MUC16* mRNA levels were significantly increased in 48.8% (41/84) of the NSCLC tissues (Table 1). This result demonstrates that increased *MUC16* expression is associated with cancerous tissue. However, *MUC16* mRNA expression did not correlate with gender (*p* = 0.74), age (*p* = 0.27), or histology type (*p* = 0.53). Interestingly, *MUC16* mRNA expression was found to be relatively higher in patients living in the heavily and moderately polluted regions of Xuanwei and Fuyuan (*p* < 0.05, Fisher’s exact test). Though *MUC16* up-regulation was observed in 51% of smokers, the overall *MUC16* mRNA expression was not significantly different between smokers and non-smokers (*p* > 0.05, Fisher’s exact test). In addition, statistical analysis concluded that patients who were living in heavily and moderately polluted regions and were also smokers, had higher *MUC16* mRNA levels compared to those who were living in relatively clear regions and were non-smokers (*p* < 0.05, Fisher’s exact test), indicating that air pollution may be the actual cause of *MUC16* up-regulation and that smoking could only boost *MUC16* expression in the patients residing in areas that were highly polluted. However, our data, which must be categorized as a pilot study, need to be confirmed in further studies.

**Table 1:** Association of MUC16 mRNA expression with clinical and environmental features

Features	Total No. (Pairs)	MUC16 up-regulated patients
**Total Subject**	84	41 (49%)
**Gender**		
Male	51	26 (50%)
Female	33	15 (45%)
**Age (years)**		
≤ 60	26	15 (58%)
> 60	58	26 (45%)
**Air pollution degrees**		
A	41	26 (63%)^*^
B	19	11 (57%)^*^
C	24	4 (17%)
**Smoking status**		
Ever	43	22 (51%)
Never	41	19 (46%)
**Histological types**		
AD	54	25 (46%)
SCC	30	16 (53%)

^*^P < 0.05; A: heavily polluted regions; B: moderately polluted regions; C: less polluted regions. AD: adenocarcinoma; SCC: squamous cell carcinoma.

To evaluate *MUC16* expression in cultured lung cancer cells, 14 cell lines were analyzed in this study. Three lung cancer cell lines (A549, 801-D, and NCI-H446) expressed higher *MUC16* mRNA levels compared to those of immortal human bronchial epithelial cell lines.

### MUC16 gene mutations were detected in lung cancer tissues and cell lines

In total, 22 tissue samples (10 pairs of NSCLC and their adjacent nonmalignant tissues as well as two cancerous tissues) and 10 lung cancer cell lines were selected for sequencing of the captured target gene to analyze the distribution of mutations within the *MUC16* gene. The tissues samples were divided into two groups, the *MUC16* up-regulated group and the *MUC16* unchanged/down-regulated group, based on the *MUC16* mRNA levels. Various types of mutations within the *MUC16* gene were observed (Supplementary Figure 2A). Total single-nucleotide polymorphism (SNP) and insertion and deletion (InDel) data were summarized for each individual and then compared between the two groups. After eliminating all the shared SNPs and InDels, distinct patterns were evident for the *MUC16* up-regulated group and the *MUC16* unchanged/down-regulated group. Some specific sites and regions that had a significantly unbalanced mutation distribution between the two groups were selected for further study.

**Figure 1:**
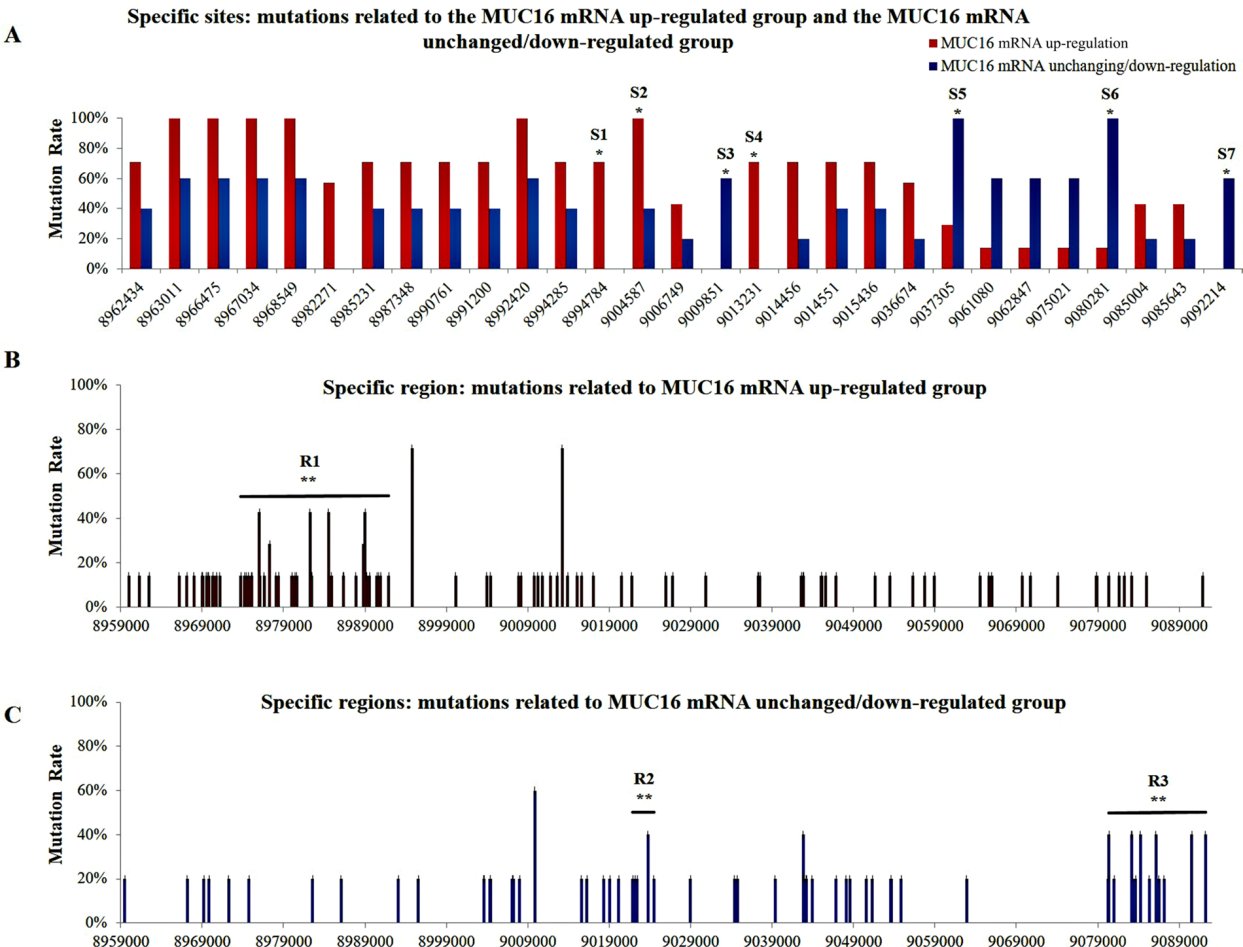
Distribution of mutations within the MUC16 gene (**A**) Mutations at specific sites in the *MUC16* up-regulated and unchanged/down-regulated groups. (**B**) More mutations occurred in region 1 (R1) in the *MUC16* up-regulated group. (**C**) Mutations occurring at region 2 (R2) and region 3 (R3) in the *MUC16* unchanged/down-regulated group. Statistical significance was calculated using Fisher’s exact test (^*^p < 0.05, ^**^p < 0.01).

Of the 12 sets of NSCLC tissues obtained from patients residing in air-polluted regions (Xuanwei and Fuyuan), seven and five cases showed up-regulated *MUC16* mRNA and unchanged/down-regulated *MUC16* mRNA, respectively. Overall, 561 mutation sites within the *MUC16* gene were identified in both groups (the *MUC16* mRNA up-regulated group and the *MUC16* mRNA unchanged/down-regulated group). In addition to the 400 mutation sites that were evenly shared among the two groups, 97 and 64 mutation sites were different in the *MUC16* up-regulated group and unchanged/down-regulated group, respectively (Supplementary Table 6). A statistically significant difference in the intron mutation rate was observed between the *MUC16* up-regulated and unchanged/down-regulated groups (Supplementary Figure 2B). Certain mutation focal points identified within the *MUC16* gene displayed an unbalanced mutation rate between the *MUC16* up-regulated group and the *MUC16* unchanged/down-regulated group (Figure 1A; Table 2). In region 1 (R1; GRCh37/hg19 coordination: 8973772-8991939), which covers nine small exons and most of the introns, 42 mutation points were found in the *MUC16* up-regulated group, whereas only three mutations were reported in R1 in the *MUC16* unchanged/down-regulated group. There were significant differences in the mutation rates in R1 between the *MUC16* up-regulated group and the *MUC16* unchanged/down-regulated group (*p* < 0.01, Fisher’s exact test) (Figure 1B). Additionally, six mutations were detected in region 2 (R2; GRCh37/hg19 coordination: 9021885-9024488) which spans two exons and most introns, in the *MUC16* unchanged/down-regulated group (*p* < 0.01, Fisher’s exact test), compared to no mutations in R2 being observed in the *MUC16* up-regulated group (Figure 1C). In region 3 (R3; GRCh37/hg19 coordination: 9080357-9092214), which covers some of the promoter region and the first and second exon (Figure 1C), the mutation rates were statistically different between the *MUC16* up-regulated group and the *MUC16* unchanged/down-regulated group (*p* < 0.01, Fisher’s exact test). Detailed results from these specific regions are listed in Table 2.

**Table 2:** Relationships between *MUC16* gene mutations at the specific sites and regions as well as *MUC16* mRNA expression

Specific-sites /Regions	Functional areas	Mutation types	DbSNP141	Positions	Total numbers of mutations^*^		*P*-value
					MUC16 up-regulated tissues (7)^	MUC16 unchanged/ down-regulated tissues (5)^	
S1	Intron	SNP	rs62120176	8994784	5^1^	0	0.028
S2	Intron	SNP	rs55650349	9004587	7^1^	2^1^	0.045
S3	Intron	SNP	rs75371087	9009851	0	3^1^	0.045
S4	Intron	SNP	rs35107941	9013231	5^1^	0	0.028
S5	Intron	InDel	rs34059802	9037305	2^1^	5^1^	0.028
S6	Intron	InDel	Novel	9080281	1^1^	5^1^	0.015
S7	Up-stream	InDel	rs35428697	9092214	0	3^1^	0.045
R1	Exon and intron	InDel, SNP	Novel	8991939-8973772	42^2^	3^2^	0
R2	Exon and intron	InDel, SNP	Novel	9024488-9021885	0	6^2^	0.002
R3	Promoter region and exon	InDel, SNP	Novel	9092214-9080357	7^2^	22^2^	0

^*^Accumulated numbers of mutations for a specific site and a region in the 7 cases of the *MUC16* up-regulated tissue or the 5 cases of the *MUC16* unchanged/down-regulated tissue; ^Numbers of cases examined in parentheses; S: specific site; R: specific region; SNP: single-nucleotide polymorphism; InDel: insertion and deletion; ^1^Total numbers of mutation at a specific site. [Note: one mutation may occur at a specific site in a patient]; ^2^Total numbers of mutations at a specific region. [Note: multiple mutations may occur at a specific region in a patient].

We noticed that non-synonymous mutation within the *MUC16* gene were detected more frequently in air pollution-related lung cancer in the present study compared with other lung cancer data from the cBioPortal (http://cbioportal.org) [15]. In our previous study, whole genome sequencing also revealed that some genes whose mutation rates and numbers in the lung cancer of highly polluted regions were significantly higher than in NSCLCs of control regions [6]. In addition, non-synonymous mutations were frequently observed in the peripheral blood cell DNA of familial lung cancer samples obtained from air-polluted regions (Xuanwei and Fuyuan) in our current study [16]. The reason for these phenomena has not yet been completely explained. We estimate that these may be associated with long-term exposure to high pollution. All of air pollution-related lung cancer patients included in our studies lived in air-polluted areas (Xuanwei and Fuyuan) for more than 30 years.

The statistical relevance between the *MUC16* gene mutations and the degree of air pollution was evaluated by calculating *p*-values. Analysis showed that the heavily polluted areas (A) were linked to higher mutation rates in the intron (Supplementary Figure 2C). These results indicate that air pollution may play an active role in destabilizing the *MUC16* gene.

The sequencing data also revealed various mutations in the ten cell lines; however, the mutation distribution patterns were different from those in the tissue samples. Mutations that were identified at several of the specific sites and regions (e.g., S1, S2, S4, S5, and R1) in the lung cancer tissue samples were not prevalent in the cell lines.

### MUC16 mRNA was up-regulated after CRISPR/Cas9 gene editing in cultured cells

To further study the relationship between the *MUC16* up-regulation and gene mutation, the *MUC16* gene was artificially mutated by introducing sgRNA vectors into the genome in cultured cells. One region (R1) and four specific sites (S1, S2, S4, and S5) were selected for this study. A total of 42 mutation points were found in R1 in the seven NSCLC cases in which *MUC16* mRNA was up-regulated, but only three mutation points were found in the five cases in which the *MUC16* mRNA was unchanged or down-regulated. The mutation frequencies of the four specific sites in the seven NSCLC tissues in which *MUC16* mRNA was up-regulated are as follows: 5 cases (S1), 7 (S2), 5 (S4), and 2 (S5). All 14 cell lines were transfected with the two vectors. After screening, the six cell lines with the highest transfection efficiency (293T, A549, 801-D, EPLC-32M1, GLC-82, and SPC-A1) were treated with the seven different mutation systems (S1, S2, S2-1, S4, S5, S5-1, and R1) targeting the four specific sites and one regions, respectively. Overexpressed MUC16 is resistant to cisplatin in ovarian cancer, and cisplatin can be used to select for cisplatin-resistant cell populations [17]. In the present study, cisplatin-resistant cell populations were obtained from the six transfected cell lines after cisplatin treatment with different concentrations (ranging from 0.2-10 µmole/L) and terms of treatment (short- and long-term treatment).

**Figure 2:**
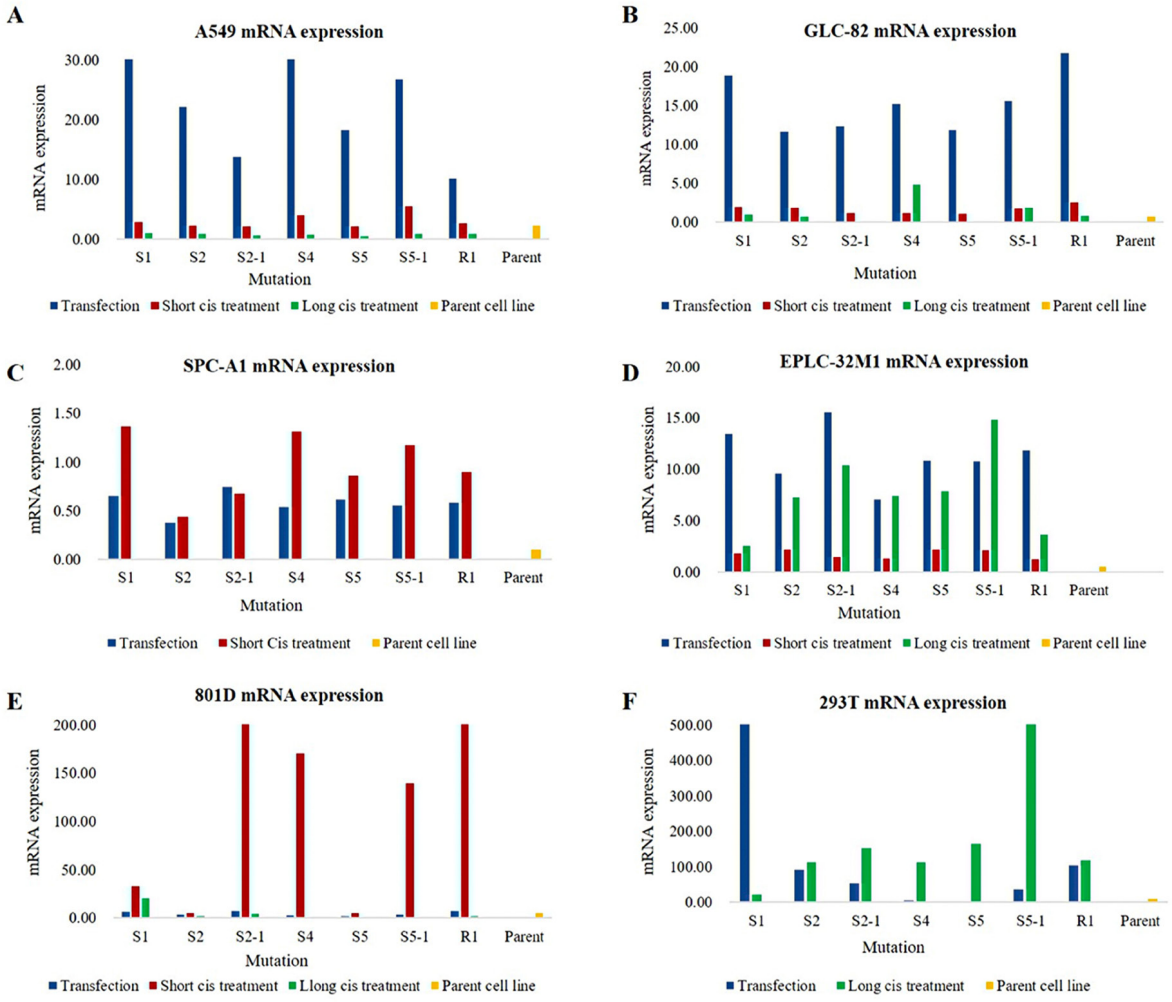
MUC16 mRNA expression in cultured cells after MUC16 gene editing (**A–F**) Increased *MUC16* mRNA levels were observed in the six cell lines after transfection alone (transfection), transfection plus short-term cisplatin treatment (Short cis treatment), and transfection plus long-term cisplatin treatment (Long cis treatment) compared to those of the parent cell lines (wild types). Cis: cisplatin. S1, S2, S2-1, S4, S5, S5-1, and R1: mutation systems used in this study.

The levels of *MUC16* mRNA were verified by qRT-PCR for the seven mutation systems in three different conditions: after transfection alone, after transfection plus short-term cisplatin treatment, and after transfection plus long-term cisplatin treatment. Different mutations within the *MUC16* gene resulted in varied expression patterns; some mutations induced unique alterations in only one cell line, whereas other mutations led to the same alteration in almost every cell line. However, compared to their respective parent cell lines (wild types), *MUC16* overexpression was observed in the six selected cell lines for all seven mutation systems (Figure 2). Although the mutation rate at S5 did not statistically correlate with *MUC16* mRNA up-regulation in the tissue samples, the cultured cells treated with the S5 and S5-1 systems showed *MUC16* mRNA up-regulation. Furthermore, the six cell lines fell into three categories according to their pattern of *MUC16* overexpression after transfection and cisplatin treatment: average increase in *MUC16* mRNA expression in A549, GLC-82, and EPLC-32M1 cells after transfection alone (without cisplatin treatment); significantly increased *MUC16* mRNA expression in SPC-A1 and 801-D cells after transfection plus short-term cisplatin treatment; and significantly increased MUC16 mRNA expression in 293T cells originating from normal human embryonic kidney cells after transfection plus long-term cisplatin treatment. These results indicate that certain mutations at specific foci (S1, S2, S4, and S5) result in *MUC16* overexpression in lung cancer cells.

### MUC16 protein was up-regulated after CRISPR/Cas9 gene editing in cultured cells

**Figure 3:**
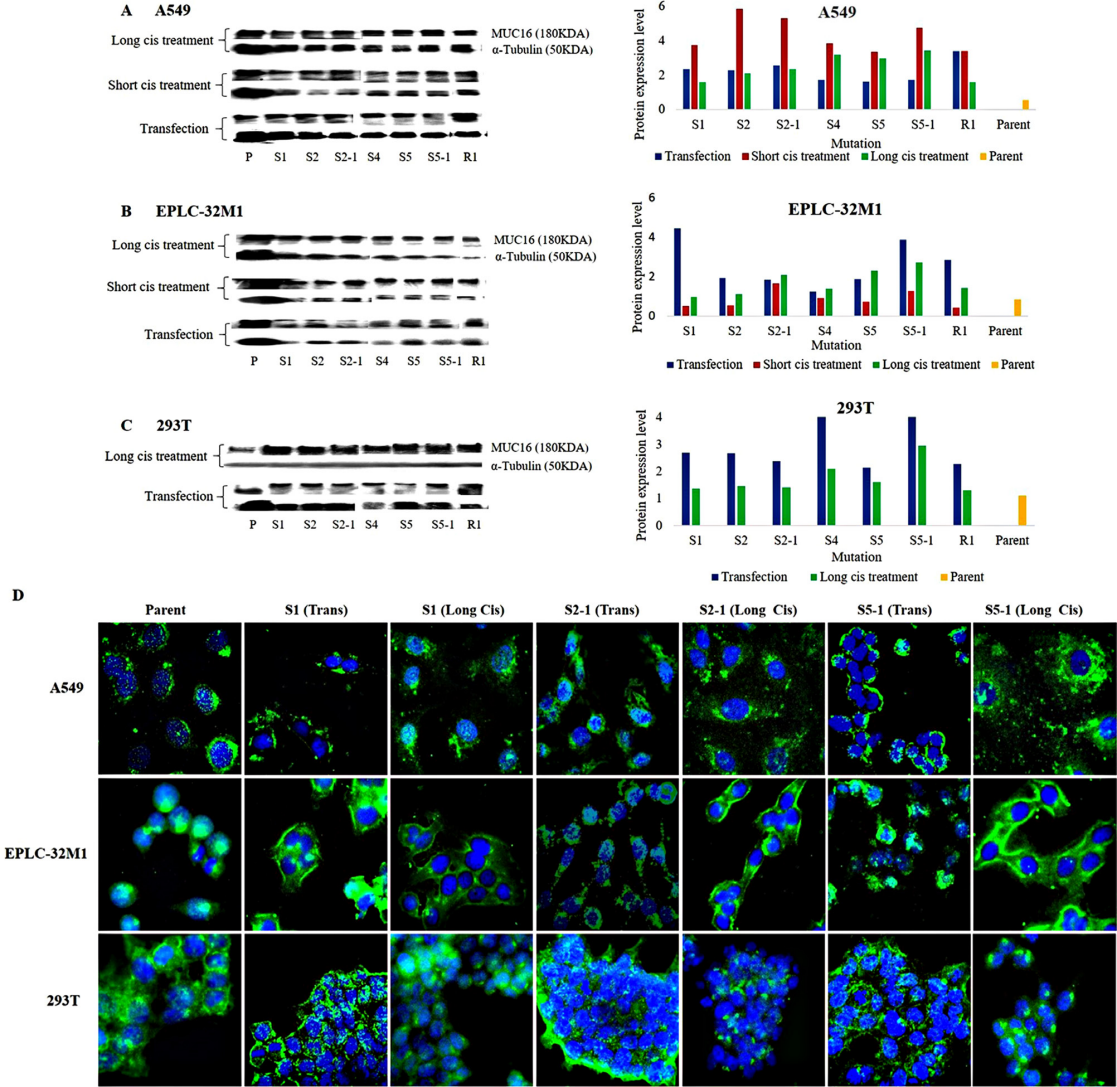
MUC16 protein expression in cultured cells after *MUC16* gene editing (**A–C**) Western blot analysis of MUC16 protein levels in the three cell lines after transfection alone (Transfection), transfection plus short-term cisplatin treatment (Short cis treatment), and transfection plus long-term cisplatin treatment (Long cis treatment). Compared to those of the parent cell lines (P and Parent), elevated protein levels were observed in cultured cells after *MUC16* gene editing. The MUC16 protein band corresponding to 180 kDa (left) was used for semi-quantitative analysis. α-Tubulin (50 kDa) was used as the internal control and reference for semi-quantitative analysis. (**D**) MUC16 protein was stained in the three cell lines using immunofluorescent antibodies. The staining signal was slightly increased at the membranes and in the cytoplasm of A549 and 293T cells after treatment compared to that of the parent cells. Note: the staining was obviously enhanced at the membranes of EPLC-32M1 cells after treatment. Parent: parent cells (wild types); Trans: transfection alone; Cis: transfection plus long-term cisplatin treatment. S1, S2, S2-1, S4, S5, S5-1, and R1: mutation systems used in this study.

To investigate changes in MUC16 protein expression induced by gene mutations, we performed western blot analysis to semi-quantitatively measure the levels of MUC16 protein in cultured cells after CRISPR/Cas9 gene editing. Western blot analyses of the three cell lines are shown in Figure 3A-C. Like the qRT-PCR results, different mutations showed varied MUC16 protein expression patterns after transfection and cisplatin treatment. Although the MUC16 protein expression was not the same as mRNA expression, overall MUC16 protein levels were elevated in the six cell lines after transfection and cisplatin treatment compared to those of their respective parent cell lines (wild types). We found that mutations at the three foci (S1, S2-1, and S5-1) were related, as similar alterations of MUC16 protein level in the 6 cell lines were observed. The same mutations at these foci were also highlighted for *MUC16* mRNA expression, indicating that these mutations may induce MUC16 protein overexpression in cultured cells.

Localization of the MUC16 protein was generally observed at the membrane and in the cytoplasm of cultured cells, as determined by immunofluorescence staining. However, MUC16 was predominantly concentrated in the Golgi apparatus and cytoplasm of EPLC-32M1 parent cells (wild types). Interestingly, MUC16 was more abundant at the membranes and in the cytoplasm of EPLC-32M1 cells after CRISPR/Cas9 gene editing. Moreover, the immunofluorescence signal for MUC16 was strengthened after treatment with almost all the mutation focus systems compared to that of their parent cells (wild types) in the six cell lines, validating the western blotting results. Immunofluorescence staining results in the three cell lines is shown in Figure 3D.

The western blot and immunofluorescence analyses confirmed that MUC16 protein levels were increased in cultured cells after CRISPR/Cas9 gene editing, which suggests that certain mutations within the *MUC16* gene may have a functionally disruptive impact on MUC16 protein expression. The stably mutated cells obtained using CRISPR/Cas9 gene editing that overexpress MUC16 protein can be used for functional experiments.

### MUC16 overexpression induced by CRISPR/Cas9 gene editing stimulated lung cancer cell proliferation

**Figure 4:**
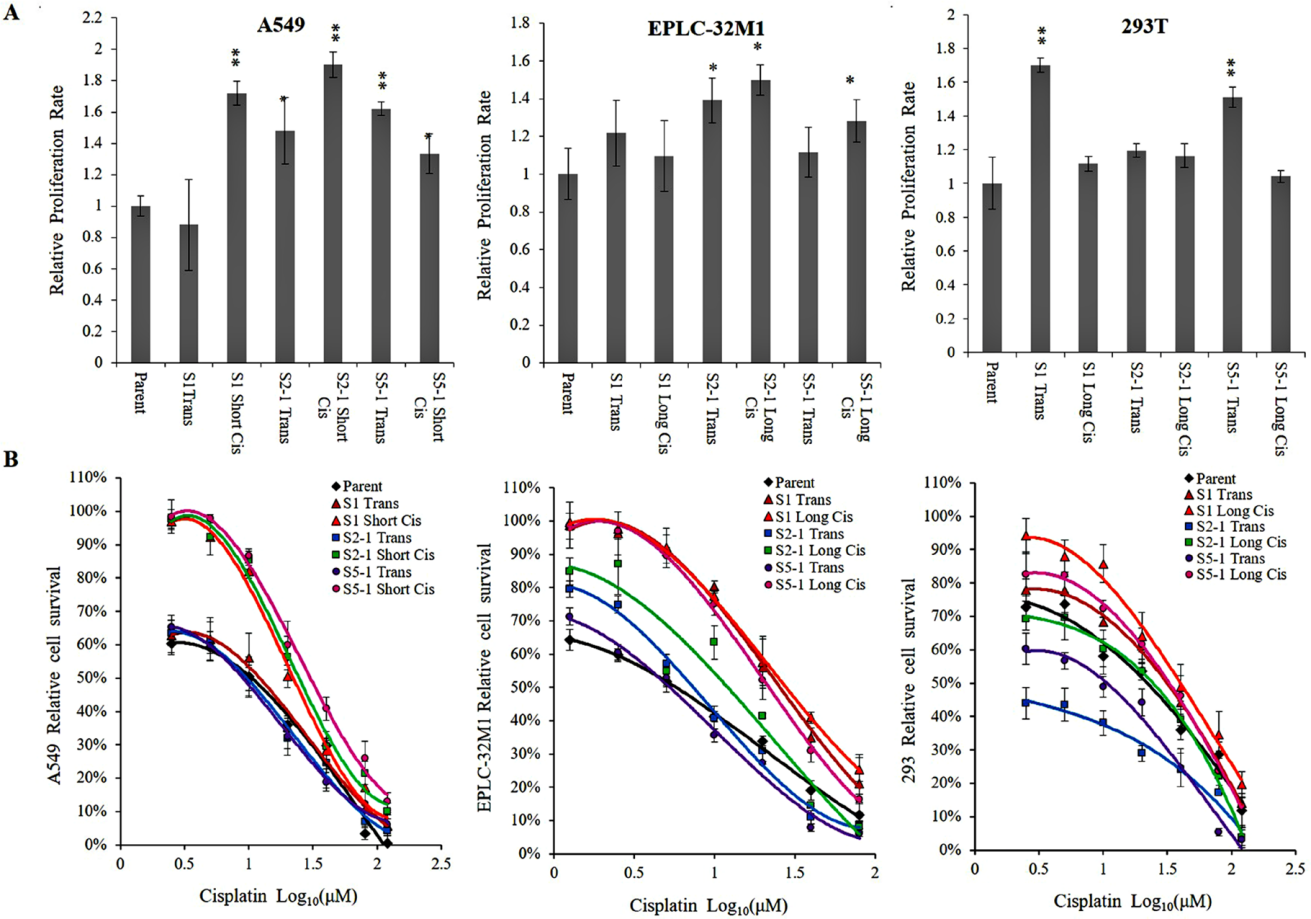
Cellular proliferation and resistance to cisplatin in cultured cells after *MUC16* gene editing (**A**) The bar graphs demonstrate a significant increase in the proliferation rate of cultured cells after treatment compared to that of the respective parent cells (Student’s *t*-test; ^*^p < 0.05, ^**^p < 0.01). (**B**) Parent cells, transfected cells, and cisplatin-resistant cell populations were incubated with different concentrations of cisplatin, and then cell viability was determined. The cell-survival curves demonstrate a significant increase in cisplatin resistance in the cultured cells after treatment compared to that of the respective parent cells. Parent: parent cells (wild types); S1, S2-1, and S5-1: mutation systems used in this study; Trans: transfection alone; Short Cis: transfection plus short-term cisplatin treatment; Long Cis: transfection plus long-term cisplatin treatment.

To further investigate the influence of *MUC16* gene mutations on cellular behavior, we selected the most important mutations at the three foci (S1, S2-1, and S5-1), and scrutinized the effects of these mutations on cell proliferation in A549, EPLC-32M1, and 293T cells. Significantly increased proliferative ability was observed in the three cell lines subjected to CRISPR/Cas9 gene editing compared with the abilities of their respective parent cells (wild types) (Figure 4A), particularly in the A549 and EPLC-32M1 cells (representative lung cancer cell lines). These results reveal that *MUC16* overexpression induced by gene mutations may promote lung cancer cell growth.

### MUC16 overexpression induced by CRISPR/Cas9 gene editing was associated with resistance to cisplatin in lung cancer cells

In the present study, the MTT assay was used to test the sensitivity of anti-cancer drugs in A549, EPLC-32M1, and 293T cells. In general, the transfected cells exhibited a higher tolerance to cisplatin compared with that of their parent cells (wild types). As expected, the cisplatin-resistant cell populations showed a stronger tolerance to cisplatin. For example, the IC50 of cisplatin in the cisplatin-resistant A549 line was approximately 23.20 ± 32.97 µM (short-term treatment), which was 3–4-fold higher than that of its parent line (7.27 µM), and the IC50 of cisplatin in the cisplatin-resistant EPLC-32M1 line was 10.38 ± 29.64 µM (long-term treatment), which was 2–7-fold higher than that of its parent line (4.66 µM). Resistance curves depicted slightly increased cisplatin survival ability in A549 and EPLC-32M1 cells (Figure 4B). These results demonstrate that *MUC16* overexpression induced by gene mutations may develop resistance to cisplatin in lung cancer cells.

In the present study, the cisplatin-resistant cell populations were exposed to azacitidine and paclitaxel, to which they were also sensitive (Supplementary Figure 3), indicating that cisplatin-resistant lung cancer cells are not tolerant towards other anti-cancer drugs, such as azacitidine and paclitaxel.

### MUC16 overexpression induced by CRISPR/Cas9 gene editing promoted the migration and invasion capacity of lung cancer cells

**Figure 5:**
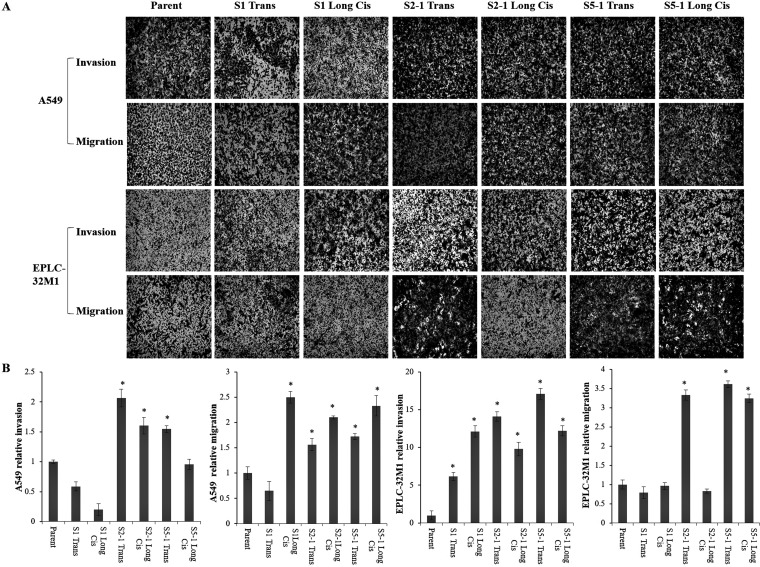
Cultured lung cancer cell invasion and migration after *MUC16* gene editing (**A**) Cell invasion and migration assays in A549 and EPLC-32M1 cells after treatment. Invading and migrating cells were photographed. (**B**) Results of the treated cells were statistically analyzed and compared with those of the parent cells. The fold changes in the numbers of invading and migrating cells are given as the means ± SD from three independent experiments (Student’s *t*-test; ^*^p < 0.05). Parent: parent cells (wild types); S1, S2-1, and S5-1: mutation systems used in this study; Tran: transfection alone; Long Cis: transfection plus long-term cisplatin treatment.

To further gain insight into whether the dissemination of lung cancer cells is related to *MUC16* overexpression and gene mutation, cellular migration and invasion were evaluated in A549 and EPLC-32M1 cells after they were transfected with the three mutation foci (S1, S2-1, and S5-1) and treated with cisplatin. Compared to those of the parent cells (wild types), *MUC16* overexpression induced by gene mutations significantly enhanced the migration and invasion efficiency of A549 and EPLC-32M1 cells (Figure 5). The ability of cell to migrate and invade is closely associated with the invasion and metastasis of cancer cells. Taken together, these findings indicate that *MUC16* overexpression induced by gene mutations may promote the invasion and metastasis of lung cancer.

## DISCUSSION

MUC16 is a trans-membrane glycoprotein that efficiently modulates cell adhesion, protein-protein interaction, and immunity by altering its expression and the nature of glycosylation [11, 18]. Our previous study demonstrated that 50% of air pollution-related lung cancers contain a mutated *MUC16* gene [6]. In a general study of lung cancer, 53% (302/572) of lung cancers, including 51% (202/394) of AD and 56% (100/178) of SCC, showed mutations in the *MUC16* gene [19]. *MUC16* was one of three genes having the highest mutation frequency across multiple cancer types [19, 20]. The high mutation frequency of *MUC16* gene was largely due to its long sequence in some cancers such as breast, liver, kidney cancer. After correcting for sequence length, *MUC16* was not ranked in the top 10 of mutated genes for these cancers. However, *MUC16* was still retained in the top 10 of mutated genes for lung and large intestine cancer after correcting for sequence length [20]. In the present study, we investigated the mRNA levels of *MUC16* in lung cancer tissue samples obtained from patients residing in air-polluted regions (Xuanwei and Fuyuan) and observed that *MUC16* mRNA was up-regulated in 48.8% (41/84) of the cancerous tissue samples compared to that of their adjacent normal tissues (*p* < 0.05). This suggests that *MUC16* mRNA up-regulation is associated with cancerous tissues. Furthermore, statistical analysis concluded that *MUC16* mRNA expression was correlated with the degree of air pollution, and patients living in heavily and moderately polluted regions who were also smokers, showed significantly increased *MUC16* mRNA expression compared to those who were living in comparatively clean areas and were non-smokers (*p* < 0.05).

In addition, we sequenced DNA from captured *MUC16* genes in air pollution-related lung cancer tissue samples. Sequencing data indicated that the mutation rate of the intron within the *MUC16* gene was significantly related to the degree of air pollution (*p* < 0.05). Several studies have reported that lung cancer in the Xuanwei population is associated with air pollution, particularly air polluted with PAHs [4–6]. PAHs, burning products of tobacco, gasoline, diesel, coal, et cetera, are the most important carcinogens resulting from smoking and air pollution. Because BaP is the major PAH index, the BaP concentration in the polluted air represented the degree of air pollution in Xuanwei and Fuyuan in this study. PAHs can directly bind DNA, form DNA adducts, and induce gene mutation [5, 21], which can result in up-regulation or down-regulation of the gene. Our results demonstrated that *MUC16* up-regulation and gene mutation occurred simultaneously in air pollution-related lung cancer, and *MUC16* up-regulation and mutation were associated with the degree of air pollution or, more precisely, the degree of PAH exposure. Moreover, PAHs are associated with C:G→A:T transversions [22, 23]. In our previous study, C:G→A:T transversions were the most frequent nucleotide substitution in lung cancer patients residing in Xuanwei and Fuyuan, as determined by whole-genome sequencing and exome sequencing [6]. In the present study, C:G→A:T transversions were also observed in the mutated *MUC16* gene (data not shown). Based on these data, we hypothesized that air pollution and PAH exposure may cause *MUC16* gene mutations, which can subsequently lead to changes in *MUC16* mRNA expression in air pollution-related lung cancer.

Interestingly, sequencing data revealed that some mutations at specific sites and regions were associated with *MUC16* mRNA up-regulation. To confirm that *MUC16* gene mutations could induce *MUC16* overexpression, we focused on mutations at four specific sites (S1, S2, S4, and S5) and one region (R1) that occurred more in lung cancer tissues in which *MUC16* mRNA was up-regulated. The CRISPR/Cas9 genome editing technique was employed to insert mutations in the six selected cell lines (A549, EPLC-32M1, GLC-82, SPC-A1, 801-D, and 293T) using seven different mutation systems (S1, S2, S2-1, S4, S5, S5-1, and R1). In general, *MUC16* expression was enhanced at both the mRNA and protein level in the cultured cells after certain mutations were induced within the *MUC16* gene using CRISPR/Cas9. Because cancer cells in which *MUC16* was overexpressed have been shown to be resistant to cisplatin in ovarian cancer [14], we also treated the cultured cells with cisplatin after the *MUC16* gene was edited, and *MUC16* overexpression induced resistance to cisplatin in the lung cancer cells. However, no significant changes in the cell-survival curves were observed after treating the cisplatin-resistant cell population with azacitidine and paclitaxel, indicating that the cisplatin-resistant cell populations still show sensitivity to azacitidine and paclitaxel. Based on the targeted gene mutation *in vitro* experiments, we proposed that certain mutations within the *MUC16* gene could induce *MUC16* overexpression and that lung cancer cells in which *MUC16* overexpression was induced by gene mutations could show resistance to cisplatin in a manner similar to ovarian cancer cells.

Finally, we investigated the effects of *MUC16* overexpression induced by gene mutations on cell growth, migration, and invasion in cultured lung cancer cells. Cellular proliferation, migration and invasion abilities were significantly increased by *MUC16* overexpression induced by mutations in the *MUC16* gene. Thus, we suggest that *MUC16* overexpression induced by gene mutations have functional impacts on cell behaviors, such as proliferation, migration, and invasion, in lung cancer. Cellular proliferation, migration, and invasion play active roles in the development and progression of cancer. Analogously, a recent study has also reported a strong association between increased *MUC16* expression and aggressiveness of cisplatin resistant lung cancer cells. Through the JAK2/STAT3/GR axis, *MUC16* overexpression down-regulates *TSPYL5*, which further mediates chemoresistance, proliferation and metastasis of lung cancer cells by suppressing p53 [24]. Additionally, although *MUC16*^c354^ transgenic animals have a normal lifespan, spontaneous tumors (including carcinoma in lung) arise at higher frequency in the double *MUC16*^c354^:*p53*^+/-^ mice compared to *p53*^+/-^ mice alone [25]. In previous studies, MUC16 up-regulation was shown to contribute to the invasion, aggression, and metastasis of tumor cells in various cancer types [12, 13, 26], particularly in ovarian cancer [27]. Moreover, MUC16 that is shed into the bloodstream can bind to certain cell types, such as natural killer (NK) cells and monocytes, to induce functional responses [18]. MUC16 up-regulation may protect cancer cells from the immune response and prevent cancer cells from cytolysis [28, 29]. Furthermore, MUC16 up-regulation has been shown to correlate with ovarian cancer relapse [30] and poor prognosis [31]. In addition to ovarian cancer, MUC16 up-regulation may be involved in the development and progression of pancreatic cancer [26]. Based on previous studies and our present study, we speculate that *MUC16* gene overexpression induced by gene mutations is not only a phenomenon, but also plays a functional role in the development and progression of lung cancer.

In conclusion, high concentrations of carcinogens in polluted air may be associated with *MUC16* gene mutations, and certain mutations (not all mutations) within the *MUC16* gene can induce *MUC16* overexpression at both the mRNA and protein level. Additionally, *MUC16* overexpression has functional impacts on the behavior of lung cancer cells, including increasing their cisplatin resistance, promoting their growth, and enhancing their migration and invasion capabilities. Thus, *MUC16* mutations potentially caused by air pollution may participate in the development and progression of air pollution-related lung cancer. In addition to ovarian cancer, MUC16 may be a candidate biomarker for air pollution-related lung cancer.

## MATERIALS AND METHODS

### Tissue samples and cell lines

A total of 84 pairs of lung cancer tissue samples were collected during surgery, which included both cancerous tissues and adjacent nonmalignant lung tissues from the same patient. Two pathologists confirmed the histological characteristics of the tumors based on their World Health Organization classification [32]. The tumor tissues that were > 70% cancer and < 10% necrotic were selected for this study, and the matched adjacent tissues contained no cancer cells. The 84 lung cancer samples comprised 54 AD cases and 30 SCC cases. The patients were between 29 and 76 years of age (average age, 52.5 years) and included 51 men and 33 women. All patients lived in Xuanwei City and Fuyuan County in Yunnan Province, China. Similar to previous studies [5, 6, 33], the entire area was further divided into three regions, heavily polluted, moderately polluted, and less polluted, according to the concentration of indoor and outdoor benzo(a)pyrene (BaP), which is the major index of PAH carcinogenicity (Supplementary Figure 1). Patient information is listed in Supplementary Table 1. This study was approved by the Ethics Committee for Human Medicine Research at Yunnan Tumor Hospital and the Kunming Institute of Zoology at the Chinese Academy of Sciences.

Fourteen cell lines (eleven lung cancer cell lines, two immortal human bronchial epithelial cell lines, and one human embryonic kidney cell line) were included in the study. The cells were cultured in either RPMI-1640 or DMEM supplemented with 10% fetal bovine serum (FBS) according to a standard protocol and maintained in an incubator at 37°C with 5% CO_2_. The cell line information is provided in Supplementary Table 2.

### Quantitative real-time polymerase chain reaction (qRT-PCR)

Total RNA was extracted from frozen tissue samples and cultured cells using TRIzol (Invitrogen, Carlsbad, CA, USA) and the phenol-chloroform extraction standard method, and cDNA was synthesized using a reverse-transcription kit (Promega, Madison, WI, USA) according to the manufacturer’s instructions. RT-PCR was performed using the StepOne Real-time PCR System (Applied Biosystems, Foster City, CA, USA) with SYBR Green (Invitrogen). The level of *MUC16* mRNA was normalized to that of glyceraldehyde-3-phosphate dehydrogenase (GAPDH) using the comparative threshold cycle number (Ct) method where ∆∆Ct_=_ ∆Ct (Target) - ∆Ct (Control). Changes in the *MUC16* mRNA levels (up-regulated, down-regulated, or unchanged) were determined using the 2^-∆∆Ct^ method as described by Livak [34]. The amplification products were analyzed using melting curve analysis, and all the reactions were repeated three times to confirm the results. The primers are: *MUC16*-F (5′-GTCCCCAACAGGCACCACACCG-3′), *MUC16*-R (5′-GGGCACTGTTGCTGGACGTTGTATT-3′) [26]; GAPDH-F (5′-TGTTGCCATCAATGACCCCTT-3′), GAPDH-R (5′-CTCCACGACGTACTCAGCG-3′).

### Western blot analysis

Cells were lysed in RIPA buffer (Solarbio, Beijing, China) supplemented with protease inhibitors (Sigma, St. Louis, MO, USA). Proteins were subjected to 6% sodium dodecyl sulfate-polyacrylamide gel electrophoresis (SDS-PAGE), and transferred onto PVDF membranes (Millipore, Bedford, MA, USA). After being blocked with 5% bovine serum albumin (BSA), the membranes were probed for MUC16; α-tubulin was used as a loading control. Mouse anti-human MUC16 monoclonal antibody (mAb) (stock: 764 µg/ml dilution factor 1:1000) and mouse anti-human α-tubulin mAb (diluted 1:2000) (Santa Cruz Biotechnology, Santa Cruz, CA, USA) were the primary antibodies used. After being incubated with peroxidase-labelled goat anti-mouse IgG [H+L] (Kirkegaard & Perry Lab, Gaithersburg, MD, USA), the proteins were detected using a chemiluminescent peroxidase substrate (Millipore). The blot X-ray films were processed with ImageJ software (NIH, Bethesda, MD, USA) for semi-quantitative measurements.

### Immunofluorescence staining

Cells were fixed with methanol and washed with phosphate buffered saline (PBS). After being blocked with 1% BSA, the cells were incubated with the primary antibody (Stock: 764 µg/ml; dilution factor 1:500) (mouse anti-human MUC16 mAb; Santa Cruz Biotechnology) at 4°C overnight. The next day, the cells were washed and incubated with two secondary antibodies (dilution factor 1:1000) (FITC/Cy3-labeled goat anti-mouse IgG; Kirkegaard & Perry Lab) separately to detect both green and red fluorescent signals. The nuclei were counterstained with Hoechst 33258 (Sigma). Images were acquired with a confocal microscope (Nikon, Tokyo, Japan).

### Target gene sequencing and specific mutation sites selection

Genomic DNA samples from tissues (ten pairs of lung cancer tissues and their adjacent nonmalignant tissues as well as two cancerous tissues) and ten cancer cell lines (all lung cancer cells except for the 95-D line) were used for sequencing of the captured target gene. Genomic DNA was prepared according to the guidelines of the International Cancer Genome Consortium (http://www.icgc.org/policies). *MUC16* gene capture and sequencing were performed by the BGI-Tech Company (Shenzhen China; http://www.genomics.cn). The total DNA available for all samples was fragmented to an average insert size of 145 bp (range 75–300 bp) and subjected to Illumina DNA sequencing library preparation. The *MUC16* gene was captured using the Agilent Sure Select Capture System (Agilent Technologies, Santa Clara, CA, USA), and DNA sequencing was performed using the Illumina HiSeq 2000/2500 platform. Each sample was sequenced at the mean depth of 140× (54.8× – 261×) coverage. Sequencing reads were aligned to the NCBI-built 37 human genome. Somatic mutations of the *MUC16* gene were identified through standard bioinformatics (*in silico*) analyses by the BGI-Tech Company. Data analysis and sequence alignment was performed to point out all the novel and known mutation in the *MUC16* gene by using different online tools and software followed by statistical analysis.

### Vector construction for editing the MUC16 gene

CRISPR/Cas9 vectors were constructed for targeting the selected specific sites and regions within the *MUC16* gene. All sgRNAs were designed using CRISPRdirect (http://crispr.dbcls.jp/). The sgRNA oligomers were synthesized and cloned into the pSpCas9(BB)-2A-GFP (PX458) vector (Addgene plasmid ID 48138). The sgRNAs were cloned by annealing two DNA oligos and ligating into a BbiI-digested vectors as previously described [35]. To improve the promoter efficiency, we added an extra 5′ G nucleotide to all of the sgRNAs that did not start with a 5′ G. The plasmid containing CRISPR/Cas9 target site for each target mutation was confirmed by plasmid DNA sequencing. A mixture of 1 μg of PX458 plasmid DNA containing each target sgRNA sequence was used for cultured cell transfection. A total of 17 sgRNA vectors targeting four specific sites and one region were built. All the sgRNA sequences are presented in Supplementary Table 3. All 17 vectors were further divided and organized into seven different groups: four specific sites (S1, S2, S4, and S5) each targeted by one vector that induced a single cut at the site (including four vectors); two specific sites (S2-1 and S5-1) each targeted by two different vectors that made double cuts upstream and downstream of the site (deleted 50-260 bp; including four vectors); and region 1 (R1) targeted by nine vectors that caused multiple cuts spanning 18 kb.

### Vector transfection

Cultured cells were first seeded in 6-well plates and then transfected using Lipofectamine 2000 (Invitrogen) when they reached a density of 70% following the manufacturer’s instructions. Two plasmids (pSpCas9(BB)-2A-GFP and Phage-to-dCas9-3XmCherry expressing green and red fluorescence, respectively) were used to evaluate transfection efficiency. Each cell line was transfected with the two plasmids separately. After the cells were incubated for an additional 48 hours (hr), they were analyzed on a FACScan flow cytometer (BD Biosciences, San Jose, CA, USA). All 14 cell lines transfected with vectors were screened to select for cell lines that had a high transfection rate. The empty vectors were used as an internal control.

### Selection of cisplatin-resistant cell populations

Cancer cells that overexpress MUC16 are resistant to cisplatin in ovarian cancer, and cisplatin has been used for MUC16-selective modulation [12]. Thus, after the selected cell lines were transfected with the constructed vectors of the seven different groups, they were maintained in culture medium containing cisplatin (Sigma) to obtain cisplatin-resistant cell populations for further study. Through cisplatin treatment, the transfected cell populations that expressed MUC16 survived, and the untransfected cell populations that did not express MUC16 were killed by cisplatin. The treatment details, including dosage and time, are listed in Supplementary Table 4. After being selected, the cells were cultured without cisplatin.

### Cell proliferation and cytotoxicity assay

Cell proliferation was analyzed using the 3-(4,5-dimethylthiazol-2yl)-2,5-diphenyltetrazolium bromide (MTT) assay. After 24, 48 and 72 hr of treatment, the MTT reagent (Sigma) was added, and the mixture was incubated for 3 hr at 37°C. Dimethyl sulfoxide was then added, and the absorbance was measured at 595 nm by a microplate reader (Bio-Rad, Hercules, CA, USA).

Cisplatin, azacitidine, and paclitaxel were purchased from Sigma and dissolved in 0.1 M NaCl solution. The aliquots were stored at –20°C and thawed immediately prior to use. For the cytotoxicity assay, cells were seeded at a density of 20,000 cells per well in 96-well plates. The next day, fixed doses of the drugs were added, and the mixtures were incubated for an additional 72 hr. The drug concentrations are listed in Supplementary Table 5. Cell viability was estimated by the MTT assay, and the percentage of cell survival was defined as the relative absorbance of the treated cells versus the untreated cells. All assays were repeated three times.

### Migration and invasion assay

For the migration assay, trans-well inserts (pore size, 8 μm; Millipore) were first incubated at 37°C in a CO_2_ incubator for 1 hr. For the invasion assay, the same inserts were first coated with Matrigel (BD Bioscience). Next, either DMEM or RPMI 1640 supplemented with 10% FBS was added into the lower chamber, and cells in serum-free DMEM or RPMI 1640 were placed into the upper chamber. The cells were allowed to migrate for 24 hr, and cells on the inserts were then fixed with methanol and stained with crystal violet. The non-migrated cells on the upper side of the chamber were removed. The insert membranes were scanned and analyzed using NIH image software (https://imagej.nih.gov/ij/), and the cell density is expressed as pixel intensity.

### Statistical analysis

All statistical analyses were conducted using SPSS 17 software (SPSS, Chicago, IL, USA). Measurement data were evaluated by the Student’s *t*-test, and enumeration data were analyzed using the chi-squared test or the Fisher’s exact test. Differences were considered significant at ^*^p < 0.05; ^**^p < 0.01.

## SUPPLEMENTARY MATERIALS FIGURES AND TABLES








