# Supplementary material for: MUC16 overexpression induced by gene mutations promotes lung cancer cell growth and invasion
Source: Oncotarget. 2018 Jan 12;9(15):12226–39. doi: 10.18632/oncotarget.24203 (PMC5844741; doi:10.18632/oncotarget.24203)
Supplement: Supplementary file 1 [file oncotarget-09-12226-s001.pdf]

MUC16 overexpression induced by gene mutations promotes lung cancer cell growth and invasion

SUPPLEMENTARY MATERIALS

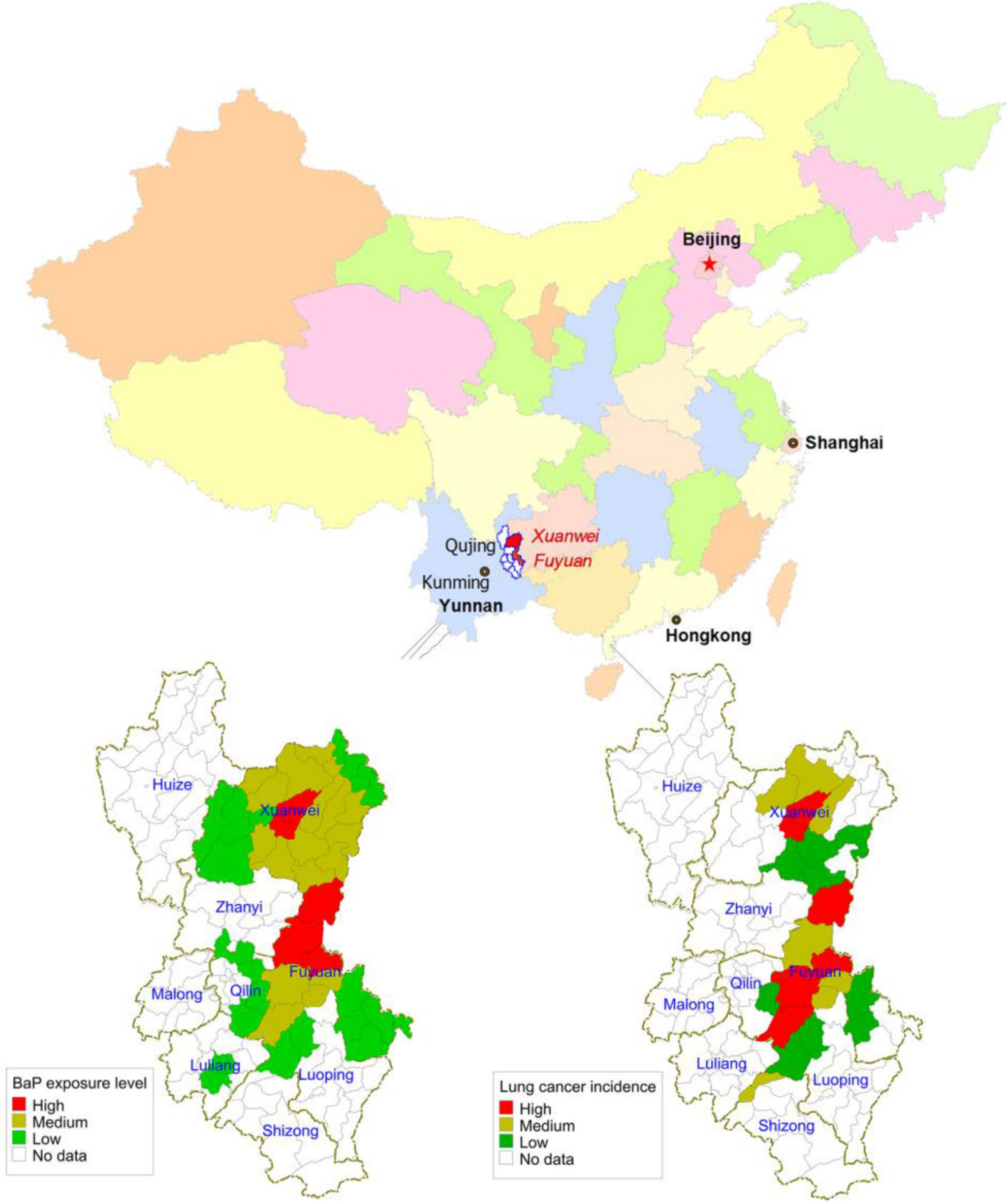

Supplementary Figure 1: BaP exposure levels and lung cancer incidence in Xuanwei and Fuyuan. Both Xuanwei and Fuyuan are located in the northeast region of Yunnan Province, China. Xuanwei and Fuyuan are rich in smoky coal mines. The high levels of indoor and outdoor air pollution are caused by burning smoky coal. BaP exposure levels are divided into three categories: high (**A**) BaP exposure levels: > 170 ng/m^3), medium (**B**) BaP exposure levels: 60~170 ng/m^3), and low (**C**) < 60 ng/m^3). The incidence of lung cancer is strongly correlated with the BaP exposure levels.

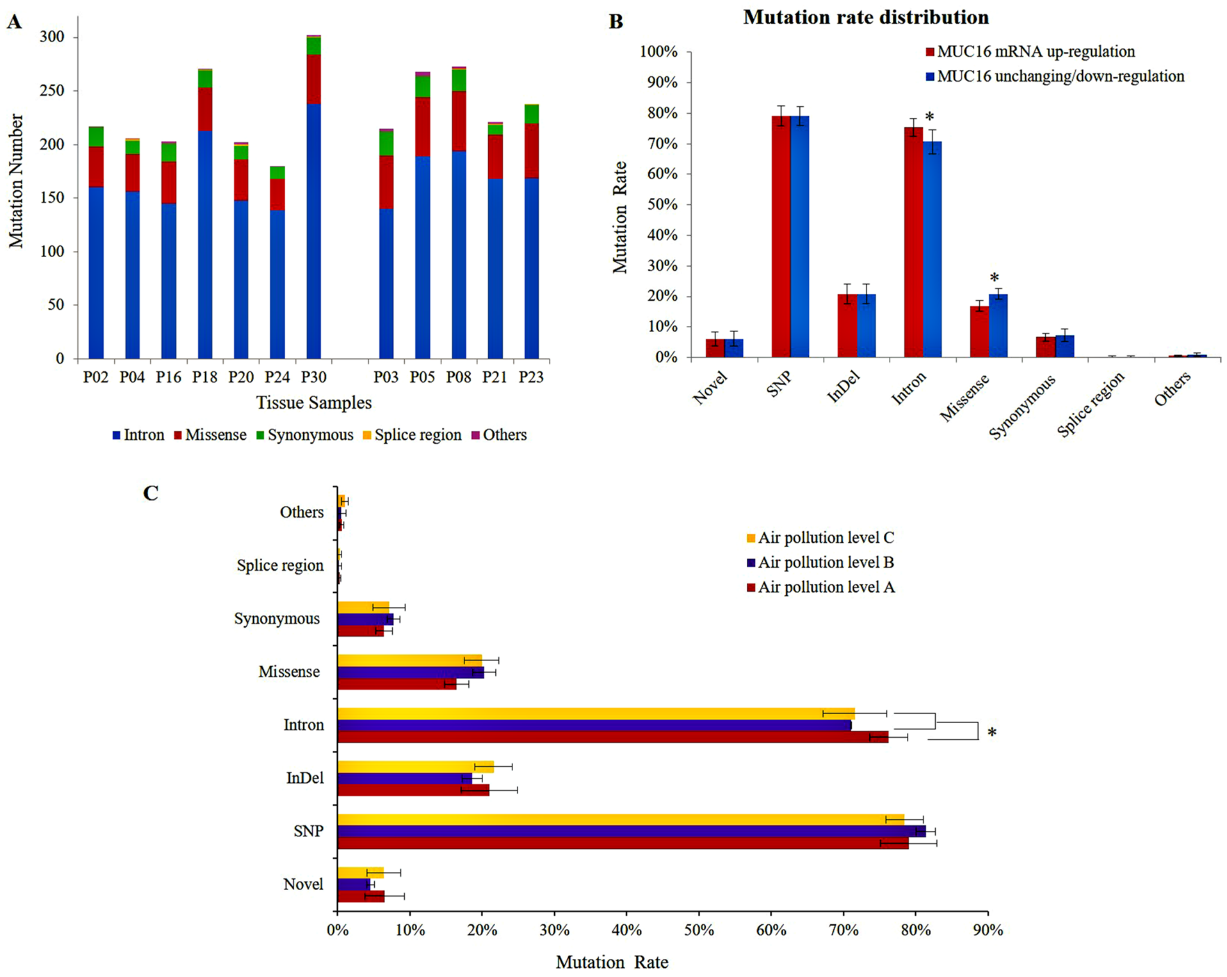

Supplementary Figure 2: Detailed information for various types of *MUC16* gene mutations. (**A**) Various types of mutations within the *MUC16* gene were identified in the 12 samples of lung cancer from patients residing in air-polluted regions (Xuanwei and Fuyuan). (**B**) Relationships between the types of *MUC16* gene mutations and *MUC16* mRNA expression. Note: the mutation rate of the intron was statistically different between the *MUC16* mRNA up-regulated group and the *MUC16* mRNA unchanged/down-regulated group. (**C**) Relationships between the types of *MUC16* gene mutations and the degree of air pollution. Note: the mutation rate of the intron was correlated with a high degree of air pollution. Air pollution level A: heavily polluted region; Air pollution level B: moderately polluted region; Air pollution level C: less polluted region. (Fisher's exact test, $^{*}p < 0.05$).

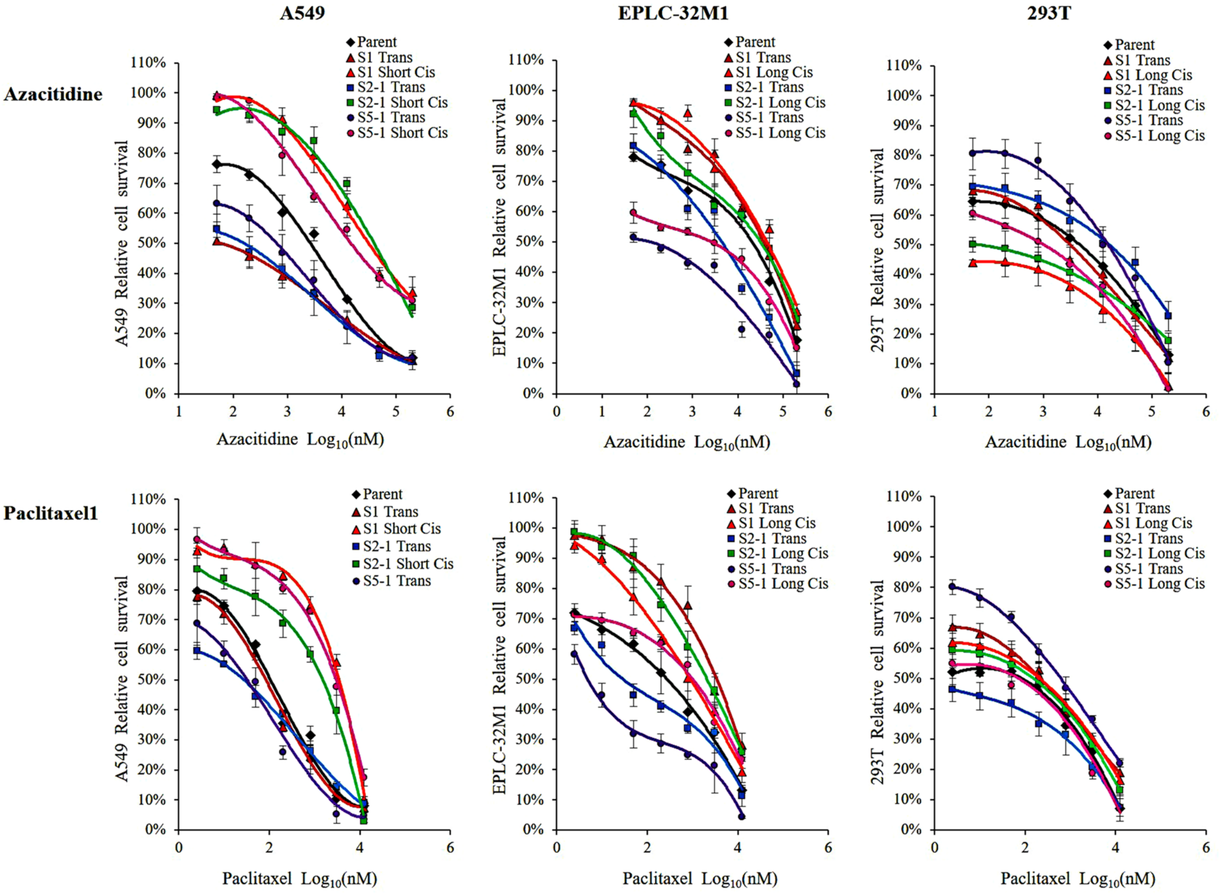

Supplementary Figure 3: Azacitidine and paclitaxel reactivity in cultured cells after *MUC16* gene editing. Parent cells, transfected cells, and cisplatin-resistant cell populations were incubated with different concentrations of azacitidine and paclitaxel, and then cell viability was determined. The cell-survival curves demonstrate no significant changes in the resistance to azacitidine and paclitaxel in the cultured cells after treatment compared to that of their respective parent cells. Note: the cisplatin-resistant cell populations were also sensitive to azacitidine and paclitaxel. Parent: parent cells (wild types); S1, S2-1, and S5-1: mutation systems used in this study; Trans: transfection alone; Short Cis: transfection plus short-term cisplatin treatment; Long Cis: transfection plus long-term cisplatin treatment.

Supplementary Table 1: Basic information of lung cancer patients included in the present study.
See Supplementary_Table_1

Supplementary Table 2: The cell lines used in this study

Name	Cell type	Culture media	Resource
EPLC-32M1	Lung squamous cell carcinoma	RPMI 10%FBS	German Cancer Research Center
SPC-A-1	Lung adenocarcinoma	RPMI 10%FBS	Cell Bank of Chinese Academy of Science
A549	Lung adenocarcinoma	RPMI 10%FBS	American Type Culture Collection
GLC-82	Lung adenocarcinoma	RPMI 10%FBS	Cell Bank of Chinese Academy of Science
XLA-07	Lung adenocarcinoma	RPMI 10%FBS	Kunming Medical College, Kunming, China
XL-JT	Lung adenocarcinoma	RPMI 10%FBS	Kunming Medical College, Kunming, China
801D	Large cell lung cancer	RPMI 10%FBS	Cell Bank of Chinese Academy of Science
NCI-H460	Large cell lung carcinoma	RPMI 10%FBS	Gifted by Prof. Zhou Guang Biao, Beijing
95-D	Large cell lung carcinoma	RPMI 10%FBS	Gifted by Prof. Zhou Guang Biao, Beijing
NCI-H292	Mucoepidermoid lung Carcinoma	RPMI 10%FBS	American Type Culture Collection
NCI-H446	Small cell lung cancer	RPMI 10%FBS	American Type Culture Collection
16HBE	Immortalized human bronchial epithelial cell	DMEM 10%FBS	Cell Bank of the Peking Union Medical College
Beas-2B	Immortalized human bronchial epithelial cell	DMEM 10%FBS	American Type Culture Collection
293T	Human embryonic kidney cells	DMEM 10%FBS	American Type Culture Collection

Supplementary Table 3: *MUC16* sgRNA oligonucleotides

Targets	sgRNA Sequence (5′-3′)	Single/Double cut
Specific 1F	CACCGATGATGGTGGTGATAACAA	Single cut
Specific 1R	AAACTTGTTATCACCACCATCATC	
Specific 2F	CACCGATGATGATGATGATGGGGG	Single cut
Specific 2R	AAACCCCCCATCATCATCATCATC	
Specific 2-1F	CACCGATTGTGATGATGATGATGAT	Double cut
Specific 2-1R	AAACATCATCATCATCATCACAATC	
Specific 2-2F	CACCGGCGGTGATGATGATTGCAG	
Specific 2-2R	AAACCTGCAATCATCATCACCGCC	
Specific 4F	CACCGTGTGGTGATGATAATGTTCG	Single cut
Specific 4R	AAACCGAACATTATCATCACCACAC	
Specific 5F	CACCGAAAAAAAAAAAGACGGAGGT	Single cut
Specific 5R	AAACACCTCCGTCTTTTTTTTTTTC	
Specific 5-1F	CACCGCCAGCCTGGGCAATGGAGCT	Double cut
Specific 5-1R	AAACAGCTCCATTGCCCAGGCTGGC	
Specific 5-2F	AAACAACCCTTAACCCTGCCTTGTC	
Specific 5-2R	CACCGACAAGGCAGGGTTAAGGGTT	
Region 1-1F	CACCGCAGCCTACCCGGCTCTAGCA	Single cut
Region 1-1R	AAACTGCTAGAGCCGGGTAGGCTGC	
Region 1-2F	AAACGGAGCGGTGTAGGACCAATAC	Single cut
Region 1-2R	CACCGTATTGGTCCTACACCGCTCC	
Region 1-3F	CACCGTGTATAAGAGTTTCCTAGGC	Single cut
Region 1-3R	AAACGCCTAGGAAACTCTTATACAC	
Region 1-4F	CACCGCCCAGGTCTGCCTATCAAGC	Single cut
Region 1-4R	AAACGCTTGATAGGCAGACCTGGGC	
Region 1-5F	AAACCAGCAGCATGGTTTTATTTC	Single cut
Region 1-5R	CACCGAAATAAAACCATGCTGCTG	
Region 1-6F	AAACGTTGTATGCCCTTTCCTCTC	Single cut
Region 1-6R	CACCGAGAGGAAAGGGCATACAAC	
Region 1-7F	AAACTCGGTTCGCAGTACATCAGC	Single cut
Region 1-7R	CACCGCTGATGTACTGCGAACCGA	
Region 1-8F	AAACAGCGTGTGGACCTGAAATTGC	Single cut
Region 1-8R	CACCGCAATTTCAGGTCCACACGCT	
Region 1-9F	CACCGCTTCACTTCACTATGTATTC	Single cut
Region 1-9R	AAACGAATACATAGTGAAGTGAAGC	
U6-F	GAGGGCCTATTTCCCATGATTCC	Sequencing Primer
U6-R	GTTGGGCGGTCAGCCAGGCGGGC	

PAM sequences are labeled in red. The oligomers were designed by using single cuts at specific sites spanning the mutation site if there are no reported any nonspecific matching. If nonspecific matching was reported, double cuts were made to obtain more precise specific mutations.

Supplementary Table 4: Time and dosage concentrations of cisplatin treatment in the selected cell lines

Cell line	Long term cisplatin treatment		Short term cisplatin treatment	
	Cisplatin dose μM/L	No. of treatment days	Cisplatin dose μM/L	No. of treatment days
A549	10	10	1.5	2
EPLC-32M1	1	10	0.2	5
SPC-A1	-	-	1.5	2
GLC-82	1.5	10	1.5	3
801-D	2	15	1.5	3
293T	5	30	-	-

Supplementary Table 5: Drug concentrations used in the cytotoxicity assay

Cell lines	Drug conc.	Cisplatin(μM)	Paclitaxel (nM)	Azacitidine (nM)
A549	7	2.5	2.5	50
	6	5	10	200
	5	10	50	800
	4	20	200	3100
	3	40	800	12500
	2	80	3100	50000
	1	120	12500	200000
EPLC-32M1	7	1.25	2.5	50
	6	2.5	10	200
	5	5	50	800
	4	10	200	3100
	3	20	800	12500
	2	40	3100	50000
	1	80	12500	200000
293T	7	2.5	2.5	50
	6	5	10	200
	5	10	50	800
	4	20	200	3100
	3	40	800	12500
	2	80	3100	50000
	1	120	12500	200000

An incubation time of approximately 72 hours was employed for each drug at each specific concentration.

Supplementary Table 6: Mutation distribution within the *MUC16* gene.
Sheet 1: The overall mutations within the *MUC16* gene in the *MUC16* mRNA up-regulated and *MUC16* mRNA unchanged/down-regulated tissue samples. See Supplementary
Sheet 2: The specific mutations within the *MUC16* gene related to *MUC16* mRNA up-regulation and the *MUC16* mRNA unchanged/down-regulation. See Supplementary
