# Supplementary material for: MUC16 overexpression induced by gene mutations promotes lung cancer cell growth and invasion
Source: Oncotarget. 2018 Jan 12;9(15):12226–39. doi: 10.18632/oncotarget.24203 (PMC5844741; doi:10.18632/oncotarget.24203)
Supplement: Supplementary file 2 [file oncotarget-09-12226-s002.docx]

**Supplementary Table 1. Basic information of lung cancer patients included in the present study.**

| **Patient ID** | **Gender** | **Age** | **Histology** | **Smoking** | **Air pollution** | **MUC16 expression** |
| --- | --- | --- | --- | --- | --- | --- |
| P01 | M | 69 | AD | YES | A | — |
| P02^*^ | M | 67 | AD | YES | A | + |
| P03^*^ | F | 41 | AD | NO | C | — |
| P04^*^ | F | 52 | AD | NO | A | + |
| P05^*^ | F | 63 | SCC | NO | C | — |
| P06 | M | 54 | SCC | YES | C | — |
| P07 | M | 53 | AD | YES | C | — |
| P08^*^ | M | 55 | SCC | NO | C | — |
| P09 | F | 48 | AD | NO | C | — |
| P10 | F | 46 | AD | NO | C | — |
| P11 | M | 38 | AD | YES | B | — |
| P12 | F | 42 | AD | NO | B | — |
| P13 | F | 60 | AD | NO | C | + |
| P14 | M | 51 | AD | NO | A | + |
| P15 | F | 41 | AD | NO | A | + |
| P16^*^ | F | 46 | AD | NO | A | + |
| P17 | F | 56 | AD | NO | A | + |
| P18^*^ | M | 47 | SCC | YES | A | + |
| P19 | F | 65 | AD | NO | A | — |
| P20^*^ | M | 66 | AD | YES | C | + |
| P21^*^ | M | 41 | SCC | YES | B | — |
| P22 | M | 61 | SCC | YES | C | — |
| P23^*^ | F | 34 | AD | NO | C | — |
| P24^*^ | M | 61 | AD | YES | B | + |
| P25 | M | 44 | AD | YES | A | — |
| P26 | F | 43 | AD | NO | A | + |
| P27 | F | 36 | AD | NO | B | — |
| P28 | F | 48 | AD | NO | B | + |
| P29 | F | 68 | SCC | NO | A | + |
| P30^*^ | F | 41 | SCC | NO | A | + |
| P31 | M | 59 | AD | YES | A | + |
| P32 | M | 60 | SCC | YES | A | + |
| P33 | M | 70 | AD | YES | A | — |
| P34 | M | 38 | SCC | YES | B | + |
| P35 | F | 62 | AD | NO | A | + |
| P36 | M | 59 | AD | YES | C | — |
| P37 | M | 58 | SCC | YES | C | — |
| P38 | F | 40 | SCC | NO | C | — |
| P39 | M | 68 | SCC | YES | A | + |
| P40 | M | 76 | SCC | YES | A | — |
| P41 | M | 43 | AD | NO | C | + |
| P42 | M | 46 | AD | YES | A | + |
| P43 | M | 58 | SCC | YES | B | + |
| P44 | M | 49 | SCC | NO | A | — |
| P45 | M | 55 | SCC | YES | A | — |
| P46 | M | 62 | AD | YES | B | + |
| P47 | M | 42 | AD | YES | A | — |
| P48 | F | 60 | AD | NO | A | — |
| P49 | M | 48 | AD | YES | B | + |
| P50 | M | 51 | SCC | YES | C | — |
| P51 | M | 67 | SCC | YES | C | + |
| P52 | F | 50 | AD | NO | A | — |
| P53 | M | 60 | SCC | NO | A | + |
| P54 | F | 62 | SCC | NO | B | — |
| P55 | M | 50 | AD | NO | A | — |
| P56 | M | 59 | AD | YES | A | — |
| P57 | M | 55 | AD | YES | B | + |
| P58 | F | 38 | AD | YES | B | + |
| P59 | M | 50 | AD | YES | B | — |
| P60 | M | 47 | SCC | YES | A | + |
| P61 | M | 71 | AD | YES | C | — |
| P62 | F | 40 | AD | NO | C | — |
| P63 | M | 47 | SCC | YES | A | — |
| P64 | M | 54 | AD | NO | A | — |
| P65 | F | 39 | AD | NO | B | + |
| P66 | M | 63 | SCC | YES | A | + |
| P67 | M | 49 | AD | YES | A | + |
| P68 | F | 30 | AD | NO | A | + |
| P69 | F | 43 | SCC | NO | B | + |
| P70 | F | 51 | AD | NO | C | — |
| P71 | M | 42 | SCC | YES | B | + |
| P72 | M | 41 | AD | NO | C | — |
| P73 | M | 60 | SCC | YES | A | + |
| P74 | M | 70 | SCC | YES | C | + |
| P75 | M | 70 | AD | YES | B | + |
| P76 | M | 74 | AD | YES | A | — |
| P77 | F | 29 | AD | NO | A | + |
| P78 | M | 50 | SCC | YES | A | + |
| P79 | M | 38 | AD | NO | A | + |
| P80 | F | 45 | SCC | NO | C | — |
| P81 | F | 50 | AD | NO | C | — |
| P82 | M | 64 | AD | YES | A | — |
| P83 | F | 42 | AD | NO | B | — |
| P84 | F | 52 | AD | NO | A | — |

*：Samples selected for target region sequencing. +：up-regulation; —：down-regulation/no change. A: heavily polluted regions; B: moderately polluted regions; C: less polluted regions. AD: adenocarcinoma; SCC: squamous cell carcinoma.
