# Supplementary material for: MUC16 overexpression induced by gene mutations promotes lung cancer cell growth and invasion
Source: Oncotarget. 2018 Jan 12;9(15):12226–39. doi: 10.18632/oncotarget.24203 (PMC5844741; doi:10.18632/oncotarget.24203)
Supplement: Supplementary file 3 [file oncotarget-09-12226-s003.docx]

| **Supplementary Table 6: The distribution of mutations within the *MUC16* gene.** | | | | | | |  | |  |  |
| --- | --- | --- | --- | --- | --- | --- | --- | --- | --- | --- |
| **Sheet 1: The overall mutations within the *MUC16* gene in the *MUC16* mRNA up-regulated and *MUC16* mRNA unchanged/down-regulated tissue samples.** | | | | | | | |  | | |
| **Mutations in *MUC16* up-regulated tissue samples** | **Total number** | **Mutation rate** |  | **Mutations in *MUC16* down-regulated/unchanged tissue samples** | **Total number** | **Mutation rate** | |  |  |  |
| 8960007 | 1 | 0.142857143 |  | 8959496 | 1 | 0.2 | |  |  |  |
| 8960033 | 7 | 1 |  | 8960033 | 5 | 1 | |  |  |  |
| 8960310 | 7 | 1 |  | 8960310 | 5 | 1 | |  |  |  |
| 8960841 | 7 | 1 |  | 8960841 | 5 | 1 | |  |  |  |
| 8961272 | 1 | 0.142857143 |  | 8961772 | 5 | 1 | |  |  |  |
| 8961772 | 7 | 1 |  | 8961981 | 5 | 1 | |  |  |  |
| 8961981 | 7 | 1 |  | 8962315 | 2 | 0.4 | |  |  |  |
| 8962315 | 2 | 0.285714286 |  | 8962434 | 2 | 0.4 | |  |  |  |
| 8962434 | 5 | 0.714285714 |  | 8962461 | 3 | 0.6 | |  |  |  |
| 8962461 | 6 | 0.857142857 |  | 8963011 | 3 | 0.6 | |  |  |  |
| 8962489 | 1 | 0.142857143 |  | 8963898 | 1 | 0.2 | |  |  |  |
| 8963011 | 7 | 1 |  | 8963904 | 1 | 0.2 | |  |  |  |
| 8963898 | 2 | 0.285714286 |  | 8964175 | 2 | 0.4 | |  |  |  |
| 8963904 | 2 | 0.285714286 |  | 8964190 | 3 | 0.6 | |  |  |  |
| 8964175 | 3 | 0.428571429 |  | 8964959 | 2 | 0.4 | |  |  |  |
| 8964190 | 3 | 0.428571429 |  | 8966475 | 3 | 0.6 | |  |  |  |
| 8964959 | 2 | 0.285714286 |  | 8967034 | 3 | 0.6 | |  |  |  |
| 8966181 | 1 | 0.142857143 |  | 8967133 | 3 | 0.6 | |  |  |  |
| 8966475 | 7 | 1 |  | 8967182 | 1 | 0.2 | |  |  |  |
| 8967034 | 6 | 0.857142857 |  | 8967400 | 2 | 0.4 | |  |  |  |
| 8967133 | 6 | 0.857142857 |  | 8967651 | 4 | 0.8 | |  |  |  |
| 8967137 | 1 | 0.142857143 |  | 8967958 | 5 | 1 | |  |  |  |
| 8967400 | 2 | 0.285714286 |  | 8968194 | 5 | 1 | |  |  |  |
| 8967651 | 5 | 0.714285714 |  | 8968232 | 1 | 0.2 | |  |  |  |
| 8967958 | 7 | 1 |  | 8968483 | 2 | 0.4 | |  |  |  |
| 8968009 | 1 | 0.142857143 |  | 8968549 | 3 | 0.6 | |  |  |  |
| 8968194 | 7 | 1 |  | 8969206 | 1 | 0.2 | |  |  |  |
| 8968232 | 4 | 0.571428571 |  | 8969749 | 5 | 1 | |  |  |  |
| 8968483 | 2 | 0.285714286 |  | 8969859 | 1 | 0.2 | |  |  |  |
| 8968549 | 7 | 1 |  | 8969885 | 2 | 0.4 | |  |  |  |
| 8968981 | 1 | 0.142857143 |  | 8970469 | 2 | 0.4 | |  |  |  |
| 8969079 | 1 | 0.142857143 |  | 8970912 | 2 | 0.4 | |  |  |  |
| 8969553 | 1 | 0.142857143 |  | 8971185 | 2 | 0.4 | |  |  |  |
| 8969715 | 1 | 0.142857143 |  | 8971299 | 2 | 0.4 | |  |  |  |
| 8969749 | 7 | 1 |  | 8972034 | 5 | 1 | |  |  |  |
| 8969826 | 1 | 0.142857143 |  | 8972042 | 2 | 0.4 | |  |  |  |
| 8969885 | 2 | 0.285714286 |  | 8972240 | 1 | 0.2 | |  |  |  |
| 8970292 | 1 | 0.142857143 |  | 8972745 | 1 | 0.2 | |  |  |  |
| 8970357 | 1 | 0.142857143 |  | 8972918 | 2 | 0.4 | |  |  |  |
| 8970469 | 2 | 0.285714286 |  | 8972961 | 2 | 0.4 | |  |  |  |
| 8970770 | 1 | 0.142857143 |  | 8972984 | 2 | 0.4 | |  |  |  |
| 8970912 | 2 | 0.285714286 |  | 8972996 | 2 | 0.4 | |  |  |  |
| 8971185 | 3 | 0.428571429 |  | 8973217 | 2 | 0.4 | |  |  |  |
| 8971191 | 1 | 0.142857143 |  | 8973757 | 2 | 0.4 | |  |  |  |
| 8971299 | 3 | 0.428571429 |  | 8974237 | 2 | 0.4 | |  |  |  |
| 8972034 | 7 | 1 |  | 8974634 | 2 | 0.4 | |  |  |  |
| 8972042 | 2 | 0.285714286 |  | 8974745 | 1 | 0.2 | |  |  |  |
| 8972745 | 2 | 0.285714286 |  | 8975158 | 1 | 0.2 | |  |  |  |
| 8972918 | 2 | 0.285714286 |  | 8975262 | 2 | 0.4 | |  |  |  |
| 8972961 | 2 | 0.285714286 |  | 8975380 | 1 | 0.2 | |  |  |  |
| 8972984 | 2 | 0.285714286 |  | 8975600 | 1 | 0.2 | |  |  |  |
| 8972996 | 2 | 0.285714286 |  | 8975770 | 1 | 0.2 | |  |  |  |
| 8973217 | 2 | 0.285714286 |  | 8976075 | 1 | 0.2 | |  |  |  |
| 8973757 | 2 | 0.285714286 |  | 8976471 | 1 | 0.2 | |  |  |  |
| 8973772 | 1 | 0.142857143 |  | 8976504 | 1 | 0.2 | |  |  |  |
| 8974222 | 1 | 0.142857143 |  | 8976713 | 1 | 0.2 | |  |  |  |
| 8974237 | 2 | 0.285714286 |  | 8976921 | 1 | 0.2 | |  |  |  |
| 8974455 | 1 | 0.142857143 |  | 8977077 | 1 | 0.2 | |  |  |  |
| 8974634 | 2 | 0.285714286 |  | 8977514 | 1 | 0.2 | |  |  |  |
| 8974733 | 1 | 0.142857143 |  | 8978542 | 5 | 1 | |  |  |  |
| 8975070 | 1 | 0.142857143 |  | 8978857 | 3 | 0.6 | |  |  |  |
| 8975120 | 1 | 0.142857143 |  | 8979112 | 3 | 0.6 | |  |  |  |
| 8975158 | 3 | 0.428571429 |  | 8979667 | 3 | 0.6 | |  |  |  |
| 8975262 | 2 | 0.285714286 |  | 8979955 | 3 | 0.6 | |  |  |  |
| 8975380 | 2 | 0.285714286 |  | 8980512 | 4 | 0.8 | |  |  |  |
| 8975600 | 2 | 0.285714286 |  | 8980545 | 2 | 0.4 | |  |  |  |
| 8975770 | 2 | 0.285714286 |  | 8980716 | 4 | 0.8 | |  |  |  |
| 8976016 | 3 | 0.428571429 |  | 8980821 | 3 | 0.6 | |  |  |  |
| 8976075 | 2 | 0.285714286 |  | 8981364 | 5 | 1 | |  |  |  |
| 8976096 | 1 | 0.142857143 |  | 8981384 | 5 | 1 | |  |  |  |
| 8976471 | 2 | 0.285714286 |  | 8981454 | 5 | 1 | |  |  |  |
| 8976504 | 2 | 0.285714286 |  | 8981838 | 5 | 1 | |  |  |  |
| 8976598 | 1 | 0.142857143 |  | 8982084 | 5 | 1 | |  |  |  |
| 8976713 | 2 | 0.285714286 |  | 8982146 | 3 | 0.6 | |  |  |  |
| 8976921 | 2 | 0.285714286 |  | 8982571 | 1 | 0.2 | |  |  |  |
| 8977077 | 2 | 0.285714286 |  | 8982603 | 5 | 1 | |  |  |  |
| 8977313 | 2 | 0.285714286 |  | 8982636 | 4 | 0.8 | |  |  |  |
| 8977514 | 2 | 0.285714286 |  | 8983229 | 5 | 1 | |  |  |  |
| 8978081 | 1 | 0.142857143 |  | 8983265 | 3 | 0.6 | |  |  |  |
| 8978384 | 1 | 0.142857143 |  | 8983519 | 1 | 0.2 | |  |  |  |
| 8978409 | 1 | 0.142857143 |  | 8983545 | 5 | 1 | |  |  |  |
| 8978542 | 7 | 1 |  | 8984216 | 3 | 0.6 | |  |  |  |
| 8978857 | 4 | 0.571428571 |  | 8984259 | 3 | 0.6 | |  |  |  |
| 8979112 | 4 | 0.571428571 |  | 8984291 | 5 | 1 | |  |  |  |
| 8979667 | 4 | 0.571428571 |  | 8984359 | 1 | 0.2 | |  |  |  |
| 8979955 | 6 | 0.857142857 |  | 8985231 | 2 | 0.4 | |  |  |  |
| 8980038 | 1 | 0.142857143 |  | 8985245 | 1 | 0.2 | |  |  |  |
| 8980378 | 1 | 0.142857143 |  | 8985268 | 1 | 0.2 | |  |  |  |
| 8980512 | 3 | 0.428571429 |  | 8985343 | 1 | 0.2 | |  |  |  |
| 8980545 | 4 | 0.571428571 |  | 8985503 | 3 | 0.6 | |  |  |  |
| 8980604 | 1 | 0.142857143 |  | 8985643 | 4 | 0.8 | |  |  |  |
| 8980716 | 4 | 0.571428571 |  | 8985662 | 1 | 0.2 | |  |  |  |
| 8980821 | 4 | 0.571428571 |  | 8985885 | 3 | 0.6 | |  |  |  |
| 8981364 | 7 | 1 |  | 8985957 | 3 | 0.6 | |  |  |  |
| 8981384 | 7 | 1 |  | 8986063 | 1 | 0.2 | |  |  |  |
| 8981454 | 7 | 1 |  | 8986085 | 1 | 0.2 | |  |  |  |
| 8981838 | 7 | 1 |  | 8986529 | 5 | 1 | |  |  |  |
| 8982084 | 7 | 1 |  | 8986621 | 5 | 1 | |  |  |  |
| 8982146 | 4 | 0.571428571 |  | 8986644 | 5 | 1 | |  |  |  |
| 8982271 | 3 | 0.428571429 |  | 8986803 | 3 | 0.6 | |  |  |  |
| 8982303 | 1 | 0.142857143 |  | 8986920 | 3 | 0.6 | |  |  |  |
| 8982497 | 1 | 0.142857143 |  | 8987133 | 5 | 1 | |  |  |  |
| 8982603 | 7 | 1 |  | 8987218 | 3 | 0.6 | |  |  |  |
| 8982636 | 5 | 0.714285714 |  | 8987348 | 2 | 0.4 | |  |  |  |
| 8983229 | 6 | 0.857142857 |  | 8988041 | 5 | 1 | |  |  |  |
| 8983265 | 4 | 0.571428571 |  | 8988233 | 4 | 0.8 | |  |  |  |
| 8983519 | 3 | 0.428571429 |  | 8988424 | 5 | 1 | |  |  |  |
| 8983545 | 7 | 1 |  | 8988928 | 5 | 1 | |  |  |  |
| 8984216 | 2 | 0.285714286 |  | 8988994 | 1 | 0.2 | |  |  |  |
| 8984259 | 4 | 0.571428571 |  | 8989074 | 1 | 0.2 | |  |  |  |
| 8984291 | 7 | 1 |  | 8989095 | 3 | 0.6 | |  |  |  |
| 8984359 | 2 | 0.285714286 |  | 8989274 | 5 | 1 | |  |  |  |
| 8984511 | 3 | 0.428571429 |  | 8989305 | 5 | 1 | |  |  |  |
| 8984900 | 1 | 0.142857143 |  | 8989345 | 4 | 0.8 | |  |  |  |
| 8985231 | 5 | 0.714285714 |  | 8989694 | 3 | 0.6 | |  |  |  |
| 8985245 | 2 | 0.285714286 |  | 8989995 | 5 | 1 | |  |  |  |
| 8985268 | 2 | 0.285714286 |  | 8990253 | 1 | 0.2 | |  |  |  |
| 8985343 | 2 | 0.285714286 |  | 8990409 | 5 | 1 | |  |  |  |
| 8985503 | 4 | 0.571428571 |  | 8990761 | 2 | 0.4 | |  |  |  |
| 8985643 | 4 | 0.571428571 |  | 8991200 | 2 | 0.4 | |  |  |  |
| 8985662 | 3 | 0.428571429 |  | 8991654 | 5 | 1 | |  |  |  |
| 8985885 | 4 | 0.571428571 |  | 8992312 | 2 | 0.4 | |  |  |  |
| 8985957 | 4 | 0.571428571 |  | 8992420 | 2 | 0.4 | |  |  |  |
| 8986063 | 3 | 0.428571429 |  | 8993113 | 1 | 0.2 | |  |  |  |
| 8986327 | 1 | 0.142857143 |  | 8994113 | 5 | 1 | |  |  |  |
| 8986366 | 1 | 0.142857143 |  | 8994285 | 2 | 0.4 | |  |  |  |
| 8986529 | 7 | 1 |  | 8994373 | 5 | 1 | |  |  |  |
| 8986621 | 7 | 1 |  | 8994843 | 5 | 1 | |  |  |  |
| 8986644 | 7 | 1 |  | 8995055 | 3 | 0.6 | |  |  |  |
| 8986803 | 4 | 0.571428571 |  | 8995056 | 1 | 0.2 | |  |  |  |
| 8986920 | 4 | 0.571428571 |  | 8995065 | 3 | 0.6 | |  |  |  |
| 8987133 | 7 | 1 |  | 8995159 | 2 | 0.4 | |  |  |  |
| 8987218 | 4 | 0.571428571 |  | 8995161 | 2 | 0.4 | |  |  |  |
| 8987348 | 5 | 0.714285714 |  | 8995170 | 2 | 0.4 | |  |  |  |
| 8987863 | 1 | 0.142857143 |  | 8995197 | 1 | 0.2 | |  |  |  |
| 8988041 | 7 | 1 |  | 8995242 | 3 | 0.6 | |  |  |  |
| 8988233 | 6 | 0.857142857 |  | 8995566 | 1 | 0.2 | |  |  |  |
| 8988424 | 7 | 1 |  | 8995853 | 5 | 1 | |  |  |  |
| 8988850 | 2 | 0.285714286 |  | 8996019 | 3 | 0.6 | |  |  |  |
| 8988928 | 7 | 1 |  | 8996182 | 3 | 0.6 | |  |  |  |
| 8988994 | 4 | 0.571428571 |  | 8996695 | 3 | 0.6 | |  |  |  |
| 8989037 | 3 | 0.428571429 |  | 8997189 | 3 | 0.6 | |  |  |  |
| 8989074 | 3 | 0.428571429 |  | 8997343 | 3 | 0.6 | |  |  |  |
| 8989095 | 2 | 0.285714286 |  | 8997662 | 1 | 0.2 | |  |  |  |
| 8989249 | 1 | 0.142857143 |  | 8997680 | 3 | 0.6 | |  |  |  |
| 8989274 | 7 | 1 |  | 8998532 | 5 | 1 | |  |  |  |
| 8989305 | 7 | 1 |  | 8999311 | 2 | 0.4 | |  |  |  |
| 8989345 | 5 | 0.714285714 |  | 8999445 | 1 | 0.2 | |  |  |  |
| 8989582 | 1 | 0.142857143 |  | 8999453 | 4 | 0.8 | |  |  |  |
| 8989694 | 4 | 0.571428571 |  | 8999675 | 4 | 0.8 | |  |  |  |
| 8989995 | 7 | 1 |  | 9000064 | 4 | 0.8 | |  |  |  |
| 8990253 | 3 | 0.428571429 |  | 9000326 | 4 | 0.8 | |  |  |  |
| 8990409 | 7 | 1 |  | 9000757 | 4 | 0.8 | |  |  |  |
| 8990467 | 1 | 0.142857143 |  | 9000852 | 3 | 0.6 | |  |  |  |
| 8990707 | 1 | 0.142857143 |  | 9000911 | 3 | 0.6 | |  |  |  |
| 8990761 | 5 | 0.714285714 |  | 9001008 | 4 | 0.8 | |  |  |  |
| 8990912 | 1 | 0.142857143 |  | 9001201 | 4 | 0.8 | |  |  |  |
| 8991200 | 5 | 0.714285714 |  | 9001276 | 4 | 0.8 | |  |  |  |
| 8991654 | 7 | 1 |  | 9001570 | 4 | 0.8 | |  |  |  |
| 8991939 | 1 | 0.142857143 |  | 9001639 | 3 | 0.6 | |  |  |  |
| 8992312 | 3 | 0.428571429 |  | 9001822 | 4 | 0.8 | |  |  |  |
| 8992420 | 6 | 0.857142857 |  | 9001986 | 4 | 0.8 | |  |  |  |
| 8994113 | 7 | 1 |  | 9002113 | 4 | 0.8 | |  |  |  |
| 8994285 | 5 | 0.714285714 |  | 9002440 | 2 | 0.4 | |  |  |  |
| 8994373 | 7 | 1 |  | 9002910 | 5 | 1 | |  |  |  |
| 8994784 | 5 | 0.714285714 |  | 9003217 | 2 | 0.4 | |  |  |  |
| 8994843 | 7 | 1 |  | 9003615 | 1 | 0.2 | |  |  |  |
| 8995055 | 4 | 0.571428571 |  | 9003618 | 4 | 0.8 | |  |  |  |
| 8995056 | 1 | 0.142857143 |  | 9003640 | 1 | 0.2 | |  |  |  |
| 8995065 | 4 | 0.571428571 |  | 9003645 | 2 | 0.4 | |  |  |  |
| 8995159 | 4 | 0.571428571 |  | 9003706 | 4 | 0.8 | |  |  |  |
| 8995161 | 4 | 0.571428571 |  | 9003749 | 4 | 0.8 | |  |  |  |
| 8995170 | 4 | 0.571428571 |  | 9004019 | 4 | 0.8 | |  |  |  |
| 8995197 | 1 | 0.142857143 |  | 9004046 | 1 | 0.2 | |  |  |  |
| 8995242 | 4 | 0.571428571 |  | 9004371 | 1 | 0.2 | |  |  |  |
| 8995853 | 7 | 1 |  | 9004424 | 1 | 0.2 | |  |  |  |
| 8996019 | 4 | 0.571428571 |  | 9004563 | 2 | 0.4 | |  |  |  |
| 8996182 | 4 | 0.571428571 |  | 9004587 | 2 | 0.4 | |  |  |  |
| 8996695 | 4 | 0.571428571 |  | 9005041 | 4 | 0.8 | |  |  |  |
| 8997189 | 4 | 0.571428571 |  | 9005512 | 1 | 0.2 | |  |  |  |
| 8997343 | 4 | 0.571428571 |  | 9005674 | 1 | 0.2 | |  |  |  |
| 8997662 | 2 | 0.285714286 |  | 9005719 | 3 | 0.6 | |  |  |  |
| 8997680 | 4 | 0.571428571 |  | 9006463 | 1 | 0.2 | |  |  |  |
| 8998532 | 7 | 1 |  | 9006749 | 1 | 0.2 | |  |  |  |
| 8999311 | 2 | 0.285714286 |  | 9007089 | 1 | 0.2 | |  |  |  |
| 8999445 | 1 | 0.142857143 |  | 9007245 | 1 | 0.2 | |  |  |  |
| 8999453 | 5 | 0.714285714 |  | 9007357 | 2 | 0.4 | |  |  |  |
| 8999675 | 7 | 1 |  | 9007630 | 2 | 0.4 | |  |  |  |
| 9000064 | 7 | 1 |  | 9007748 | 4 | 0.8 | |  |  |  |
| 9000129 | 1 | 0.142857143 |  | 9008017 | 1 | 0.2 | |  |  |  |
| 9000326 | 7 | 1 |  | 9008843 | 5 | 1 | |  |  |  |
| 9000757 | 7 | 1 |  | 9009161 | 5 | 1 | |  |  |  |
| 9000852 | 7 | 1 |  | 9009851 | 3 | 0.6 | |  |  |  |
| 9000911 | 6 | 0.857142857 |  | 9011983 | 3 | 0.6 | |  |  |  |
| 9001008 | 7 | 1 |  | 9013275 | 1 | 0.2 | |  |  |  |
| 9001201 | 7 | 1 |  | 9013355 | 3 | 0.6 | |  |  |  |
| 9001276 | 7 | 1 |  | 9014405 | 2 | 0.4 | |  |  |  |
| 9001570 | 6 | 0.857142857 |  | 9014456 | 2 | 0.4 | |  |  |  |
| 9001639 | 6 | 0.857142857 |  | 9014551 | 2 | 0.4 | |  |  |  |
| 9001822 | 7 | 1 |  | 9014569 | 2 | 0.4 | |  |  |  |
| 9001986 | 7 | 1 |  | 9014932 | 3 | 0.6 | |  |  |  |
| 9002113 | 7 | 1 |  | 9015436 | 2 | 0.4 | |  |  |  |
| 9002440 | 6 | 0.857142857 |  | 9015579 | 1 | 0.2 | |  |  |  |
| 9002910 | 7 | 1 |  | 9016251 | 1 | 0.2 | |  |  |  |
| 9003217 | 2 | 0.285714286 |  | 9017910 | 2 | 0.4 | |  |  |  |
| 9003618 | 5 | 0.714285714 |  | 9018301 | 1 | 0.2 | |  |  |  |
| 9003640 | 1 | 0.142857143 |  | 9019080 | 1 | 0.2 | |  |  |  |
| 9003645 | 1 | 0.142857143 |  | 9019429 | 4 | 0.8 | |  |  |  |
| 9003706 | 6 | 0.857142857 |  | 9019605 | 5 | 1 | |  |  |  |
| 9003749 | 7 | 1 |  | 9020154 | 1 | 0.2 | |  |  |  |
| 9003962 | 1 | 0.142857143 |  | 9020185 | 1 | 0.2 | |  |  |  |
| 9004019 | 7 | 1 |  | 9020211 | 4 | 0.8 | |  |  |  |
| 9004046 | 2 | 0.285714286 |  | 9020376 | 3 | 0.6 | |  |  |  |
| 9004391 | 1 | 0.142857143 |  | 9020533 | 4 | 0.8 | |  |  |  |
| 9004563 | 5 | 0.714285714 |  | 9020666 | 5 | 1 | |  |  |  |
| 9004587 | 6 | 0.857142857 |  | 9021275 | 3 | 0.6 | |  |  |  |
| 9005041 | 7 | 1 |  | 9021328 | 5 | 1 | |  |  |  |
| 9005512 | 3 | 0.428571429 |  | 9021374 | 4 | 0.8 | |  |  |  |
| 9005674 | 4 | 0.571428571 |  | 9021451 | 5 | 1 | |  |  |  |
| 9005719 | 5 | 0.714285714 |  | 9021564 | 2 | 0.4 | |  |  |  |
| 9006463 | 2 | 0.285714286 |  | 9021730 | 2 | 0.4 | |  |  |  |
| 9006749 | 3 | 0.428571429 |  | 9021765 | 1 | 0.2 | |  |  |  |
| 9007357 | 2 | 0.285714286 |  | 9021885 | 1 | 0.2 | |  |  |  |
| 9007630 | 2 | 0.285714286 |  | 9021951 | 3 | 0.6 | |  |  |  |
| 9007748 | 2 | 0.285714286 |  | 9022145 | 3 | 0.6 | |  |  |  |
| 9007873 | 1 | 0.142857143 |  | 9022213 | 1 | 0.2 | |  |  |  |
| 9008201 | 1 | 0.142857143 |  | 9022450 | 1 | 0.2 | |  |  |  |
| 9008843 | 6 | 0.857142857 |  | 9023732 | 2 | 0.4 | |  |  |  |
| 9009161 | 4 | 0.571428571 |  | 9023866 | 5 | 1 | |  |  |  |
| 9009762 | 1 | 0.142857143 |  | 9024297 | 1 | 0.2 | |  |  |  |
| 9010222 | 1 | 0.142857143 |  | 9024488 | 1 | 0.2 | |  |  |  |
| 9010754 | 1 | 0.142857143 |  | 9024758 | 5 | 1 | |  |  |  |
| 9011781 | 1 | 0.142857143 |  | 9024871 | 1 | 0.2 | |  |  |  |
| 9011983 | 3 | 0.428571429 |  | 9024994 | 4 | 0.8 | |  |  |  |
| 9012612 | 1 | 0.142857143 |  | 9025276 | 1 | 0.2 | |  |  |  |
| 9013231 | 5 | 0.714285714 |  | 9025826 | 4 | 0.8 | |  |  |  |
| 9013275 | 2 | 0.285714286 |  | 9026257 | 1 | 0.2 | |  |  |  |
| 9013355 | 4 | 0.571428571 |  | 9026318 | 1 | 0.2 | |  |  |  |
| 9013918 | 1 | 0.142857143 |  | 9026651 | 2 | 0.4 | |  |  |  |
| 9014405 | 3 | 0.428571429 |  | 9026781 | 5 | 1 | |  |  |  |
| 9014456 | 5 | 0.714285714 |  | 9027101 | 3 | 0.6 | |  |  |  |
| 9014551 | 5 | 0.714285714 |  | 9027790 | 1 | 0.2 | |  |  |  |
| 9014569 | 5 | 0.714285714 |  | 9028373 | 4 | 0.8 | |  |  |  |
| 9014932 | 2 | 0.285714286 |  | 9028432 | 5 | 1 | |  |  |  |
| 9015049 | 1 | 0.142857143 |  | 9028976 | 1 | 0.2 | |  |  |  |
| 9015436 | 5 | 0.714285714 |  | 9031115 | 1 | 0.2 | |  |  |  |
| 9015617 | 1 | 0.142857143 |  | 9031132 | 1 | 0.2 | |  |  |  |
| 9017045 | 1 | 0.142857143 |  | 9033314 | 2 | 0.4 | |  |  |  |
| 9017910 | 3 | 0.428571429 |  | 9033490 | 1 | 0.2 | |  |  |  |
| 9019429 | 2 | 0.285714286 |  | 9033548 | 1 | 0.2 | |  |  |  |
| 9019605 | 5 | 0.714285714 |  | 9034358 | 1 | 0.2 | |  |  |  |
| 9020154 | 3 | 0.428571429 |  | 9034426 | 4 | 0.8 | |  |  |  |
| 9020211 | 6 | 0.857142857 |  | 9034446 | 5 | 1 | |  |  |  |
| 9020376 | 4 | 0.571428571 |  | 9034725 | 1 | 0.2 | |  |  |  |
| 9020533 | 3 | 0.428571429 |  | 9035172 | 4 | 0.8 | |  |  |  |
| 9020549 | 1 | 0.142857143 |  | 9036031 | 5 | 1 | |  |  |  |
| 9020666 | 6 | 0.857142857 |  | 9036674 | 3 | 0.6 | |  |  |  |
| 9021275 | 4 | 0.571428571 |  | 9036745 | 5 | 1 | |  |  |  |
| 9021328 | 5 | 0.714285714 |  | 9036879 | 3 | 0.6 | |  |  |  |
| 9021374 | 3 | 0.428571429 |  | 9037160 | 5 | 1 | |  |  |  |
| 9021451 | 6 | 0.857142857 |  | 9037305 | 5 | 1 | |  |  |  |
| 9021564 | 5 | 0.714285714 |  | 9037386 | 1 | 0.2 | |  |  |  |
| 9021730 | 3 | 0.428571429 |  | 9037496 | 2 | 0.4 | |  |  |  |
| 9021762 | 1 | 0.142857143 |  | 9037848 | 3 | 0.6 | |  |  |  |
| 9021765 | 1 | 0.142857143 |  | 9038831 | 5 | 1 | |  |  |  |
| 9021951 | 4 | 0.571428571 |  | 9038989 | 3 | 0.6 | |  |  |  |
| 9022145 | 4 | 0.571428571 |  | 9038993 | 5 | 1 | |  |  |  |
| 9023866 | 7 | 1 |  | 9039255 | 1 | 0.2 | |  |  |  |
| 9024297 | 1 | 0.142857143 |  | 9039355 | 1 | 0.2 | |  |  |  |
| 9024758 | 7 | 1 |  | 9039428 | 5 | 1 | |  |  |  |
| 9024871 | 1 | 0.142857143 |  | 9040349 | 5 | 1 | |  |  |  |
| 9024994 | 3 | 0.428571429 |  | 9040732 | 5 | 1 | |  |  |  |
| 9025276 | 1 | 0.142857143 |  | 9040933 | 4 | 0.8 | |  |  |  |
| 9025826 | 3 | 0.428571429 |  | 9040985 | 4 | 0.8 | |  |  |  |
| 9025943 | 1 | 0.142857143 |  | 9041335 | 4 | 0.8 | |  |  |  |
| 9026257 | 1 | 0.142857143 |  | 9041986 | 1 | 0.2 | |  |  |  |
| 9026318 | 1 | 0.142857143 |  | 9042018 | 1 | 0.2 | |  |  |  |
| 9026651 | 6 | 0.857142857 |  | 9042781 | 1 | 0.2 | |  |  |  |
| 9026781 | 7 | 1 |  | 9042826 | 1 | 0.2 | |  |  |  |
| 9026813 | 1 | 0.142857143 |  | 9042829 | 2 | 0.4 | |  |  |  |
| 9027101 | 4 | 0.571428571 |  | 9042836 | 1 | 0.2 | |  |  |  |
| 9027790 | 1 | 0.142857143 |  | 9042844 | 1 | 0.2 | |  |  |  |
| 9028373 | 3 | 0.428571429 |  | 9042848 | 1 | 0.2 | |  |  |  |
| 9028432 | 6 | 0.857142857 |  | 9043142 | 1 | 0.2 | |  |  |  |
| 9030824 | 1 | 0.142857143 |  | 9043183 | 1 | 0.2 | |  |  |  |
| 9031115 | 1 | 0.142857143 |  | 9043254 | 1 | 0.2 | |  |  |  |
| 9031132 | 1 | 0.142857143 |  | 9043534 | 4 | 0.8 | |  |  |  |
| 9033314 | 2 | 0.285714286 |  | 9043680 | 3 | 0.6 | |  |  |  |
| 9033490 | 1 | 0.142857143 |  | 9043775 | 2 | 0.4 | |  |  |  |
| 9033548 | 1 | 0.142857143 |  | 9043930 | 1 | 0.2 | |  |  |  |
| 9034426 | 4 | 0.571428571 |  | 9044137 | 2 | 0.4 | |  |  |  |
| 9034446 | 7 | 1 |  | 9044534 | 1 | 0.2 | |  |  |  |
| 9035172 | 6 | 0.857142857 |  | 9044900 | 3 | 0.6 | |  |  |  |
| 9036031 | 7 | 1 |  | 9044918 | 1 | 0.2 | |  |  |  |
| 9036674 | 4 | 0.571428571 |  | 9045117 | 1 | 0.2 | |  |  |  |
| 9036745 | 7 | 1 |  | 9045216 | 5 | 1 | |  |  |  |
| 9036879 | 2 | 0.285714286 |  | 9045420 | 1 | 0.2 | |  |  |  |
| 9037160 | 4 | 0.571428571 |  | 9045490 | 2 | 0.4 | |  |  |  |
| 9037305 | 2 | 0.285714286 |  | 9045518 | 3 | 0.6 | |  |  |  |
| 9037307 | 1 | 0.142857143 |  | 9046876 | 2 | 0.4 | |  |  |  |
| 9037386 | 1 | 0.142857143 |  | 9046883 | 1 | 0.2 | |  |  |  |
| 9037496 | 2 | 0.285714286 |  | 9048102 | 1 | 0.2 | |  |  |  |
| 9037524 | 1 | 0.142857143 |  | 9048320 | 2 | 0.4 | |  |  |  |
| 9037848 | 2 | 0.285714286 |  | 9048342 | 5 | 1 | |  |  |  |
| 9038831 | 7 | 1 |  | 9048421 | 3 | 0.6 | |  |  |  |
| 9038989 | 2 | 0.285714286 |  | 9048546 | 1 | 0.2 | |  |  |  |
| 9038993 | 5 | 0.714285714 |  | 9048895 | 5 | 1 | |  |  |  |
| 9039255 | 1 | 0.142857143 |  | 9049339 | 3 | 0.6 | |  |  |  |
| 9039428 | 4 | 0.571428571 |  | 9049726 | 1 | 0.2 | |  |  |  |
| 9040349 | 6 | 0.857142857 |  | 9049910 | 3 | 0.6 | |  |  |  |
| 9040732 | 4 | 0.571428571 |  | 9050570 | 1 | 0.2 | |  |  |  |
| 9040933 | 2 | 0.285714286 |  | 9050999 | 1 | 0.2 | |  |  |  |
| 9040985 | 2 | 0.285714286 |  | 9051315 | 1 | 0.2 | |  |  |  |
| 9041335 | 2 | 0.285714286 |  | 9051406 | 5 | 1 | |  |  |  |
| 9041986 | 1 | 0.142857143 |  | 9051575 | 4 | 0.8 | |  |  |  |
| 9042018 | 2 | 0.285714286 |  | 9052236 | 5 | 1 | |  |  |  |
| 9042586 | 1 | 0.142857143 |  | 9052400 | 1 | 0.2 | |  |  |  |
| 9042781 | 2 | 0.285714286 |  | 9052498 | 4 | 0.8 | |  |  |  |
| 9042836 | 1 | 0.142857143 |  | 9052840 | 5 | 1 | |  |  |  |
| 9042840 | 1 | 0.142857143 |  | 9052996 | 4 | 0.8 | |  |  |  |
| 9042844 | 1 | 0.142857143 |  | 9053596 | 1 | 0.2 | |  |  |  |
| 9042860 | 1 | 0.142857143 |  | 9053616 | 1 | 0.2 | |  |  |  |
| 9043183 | 1 | 0.142857143 |  | 9054544 | 4 | 0.8 | |  |  |  |
| 9043534 | 2 | 0.285714286 |  | 9054853 | 1 | 0.2 | |  |  |  |
| 9043680 | 2 | 0.285714286 |  | 9055325 | 5 | 1 | |  |  |  |
| 9043775 | 2 | 0.285714286 |  | 9055753 | 5 | 1 | |  |  |  |
| 9044137 | 2 | 0.285714286 |  | 9056982 | 1 | 0.2 | |  |  |  |
| 9044534 | 1 | 0.142857143 |  | 9056989 | 3 | 0.6 | |  |  |  |
| 9044900 | 2 | 0.285714286 |  | 9057408 | 5 | 1 | |  |  |  |
| 9044918 | 1 | 0.142857143 |  | 9057687 | 5 | 1 | |  |  |  |
| 9045059 | 1 | 0.142857143 |  | 9057721 | 3 | 0.6 | |  |  |  |
| 9045086 | 1 | 0.142857143 |  | 9058624 | 3 | 0.6 | |  |  |  |
| 9045117 | 2 | 0.285714286 |  | 9058907 | 5 | 1 | |  |  |  |
| 9045216 | 7 | 1 |  | 9058942 | 4 | 0.8 | |  |  |  |
| 9045420 | 1 | 0.142857143 |  | 9059159 | 5 | 1 | |  |  |  |
| 9045490 | 2 | 0.285714286 |  | 9059307 | 5 | 1 | |  |  |  |
| 9045518 | 2 | 0.285714286 |  | 9059808 | 5 | 1 | |  |  |  |
| 9045602 | 1 | 0.142857143 |  | 9060085 | 3 | 0.6 | |  |  |  |
| 9046863 | 1 | 0.142857143 |  | 9060541 | 5 | 1 | |  |  |  |
| 9046876 | 2 | 0.285714286 |  | 9060572 | 3 | 0.6 | |  |  |  |
| 9048320 | 2 | 0.285714286 |  | 9060605 | 5 | 1 | |  |  |  |
| 9048342 | 5 | 0.714285714 |  | 9060656 | 1 | 0.2 | |  |  |  |
| 9048421 | 2 | 0.285714286 |  | 9060812 | 5 | 1 | |  |  |  |
| 9048895 | 4 | 0.571428571 |  | 9061080 | 3 | 0.6 | |  |  |  |
| 9049339 | 2 | 0.285714286 |  | 9061559 | 5 | 1 | |  |  |  |
| 9049726 | 1 | 0.142857143 |  | 9062183 | 2 | 0.4 | |  |  |  |
| 9049910 | 2 | 0.285714286 |  | 9062415 | 5 | 1 | |  |  |  |
| 9050999 | 1 | 0.142857143 |  | 9062544 | 5 | 1 | |  |  |  |
| 9051406 | 7 | 1 |  | 9062847 | 3 | 0.6 | |  |  |  |
| 9051575 | 7 | 1 |  | 9062880 | 1 | 0.2 | |  |  |  |
| 9051617 | 1 | 0.142857143 |  | 9063678 | 1 | 0.2 | |  |  |  |
| 9052236 | 7 | 1 |  | 9063935 | 1 | 0.2 | |  |  |  |
| 9052400 | 1 | 0.142857143 |  | 9065203 | 5 | 1 | |  |  |  |
| 9052498 | 3 | 0.428571429 |  | 9065510 | 5 | 1 | |  |  |  |
| 9052840 | 7 | 1 |  | 9065632 | 1 | 0.2 | |  |  |  |
| 9052996 | 3 | 0.428571429 |  | 9066259 | 5 | 1 | |  |  |  |
| 9053507 | 1 | 0.142857143 |  | 9066874 | 3 | 0.6 | |  |  |  |
| 9054544 | 4 | 0.571428571 |  | 9068530 | 5 | 1 | |  |  |  |
| 9055325 | 7 | 1 |  | 9069792 | 3 | 0.6 | |  |  |  |
| 9055753 | 7 | 1 |  | 9069892 | 3 | 0.6 | |  |  |  |
| 9056323 | 1 | 0.142857143 |  | 9070079 | 5 | 1 | |  |  |  |
| 9056982 | 1 | 0.142857143 |  | 9070194 | 5 | 1 | |  |  |  |
| 9056989 | 2 | 0.285714286 |  | 9070225 | 5 | 1 | |  |  |  |
| 9057408 | 7 | 1 |  | 9070837 | 5 | 1 | |  |  |  |
| 9057687 | 7 | 1 |  | 9071763 | 5 | 1 | |  |  |  |
| 9057721 | 3 | 0.428571429 |  | 9071834 | 2 | 0.4 | |  |  |  |
| 9057780 | 1 | 0.142857143 |  | 9071924 | 3 | 0.6 | |  |  |  |
| 9058624 | 2 | 0.285714286 |  | 9072221 | 3 | 0.6 | |  |  |  |
| 9058907 | 5 | 0.714285714 |  | 9072313 | 1 | 0.2 | |  |  |  |
| 9058942 | 3 | 0.428571429 |  | 9072742 | 1 | 0.2 | |  |  |  |
| 9058958 | 1 | 0.142857143 |  | 9072875 | 3 | 0.6 | |  |  |  |
| 9059159 | 4 | 0.571428571 |  | 9072975 | 3 | 0.6 | |  |  |  |
| 9059307 | 4 | 0.571428571 |  | 9074073 | 3 | 0.6 | |  |  |  |
| 9059808 | 7 | 1 |  | 9074950 | 1 | 0.2 | |  |  |  |
| 9060085 | 2 | 0.285714286 |  | 9075021 | 3 | 0.6 | |  |  |  |
| 9060541 | 6 | 0.857142857 |  | 9075217 | 1 | 0.2 | |  |  |  |
| 9060572 | 2 | 0.285714286 |  | 9075346 | 1 | 0.2 | |  |  |  |
| 9060605 | 7 | 1 |  | 9075565 | 5 | 1 | |  |  |  |
| 9060656 | 1 | 0.142857143 |  | 9075584 | 1 | 0.2 | |  |  |  |
| 9060812 | 7 | 1 |  | 9075635 | 1 | 0.2 | |  |  |  |
| 9061080 | 1 | 0.142857143 |  | 9075737 | 5 | 1 | |  |  |  |
| 9061559 | 6 | 0.857142857 |  | 9075969 | 1 | 0.2 | |  |  |  |
| 9062183 | 2 | 0.285714286 |  | 9076083 | 1 | 0.2 | |  |  |  |
| 9062415 | 6 | 0.857142857 |  | 9076163 | 1 | 0.2 | |  |  |  |
| 9062544 | 7 | 1 |  | 9076278 | 1 | 0.2 | |  |  |  |
| 9062847 | 1 | 0.142857143 |  | 9076728 | 1 | 0.2 | |  |  |  |
| 9063678 | 1 | 0.142857143 |  | 9076858 | 1 | 0.2 | |  |  |  |
| 9063935 | 1 | 0.142857143 |  | 9076929 | 1 | 0.2 | |  |  |  |
| 9064548 | 1 | 0.142857143 |  | 9076950 | 1 | 0.2 | |  |  |  |
| 9065203 | 4 | 0.571428571 |  | 9076991 | 1 | 0.2 | |  |  |  |
| 9065510 | 4 | 0.571428571 |  | 9077060 | 1 | 0.2 | |  |  |  |
| 9065619 | 1 | 0.142857143 |  | 9077196 | 5 | 1 | |  |  |  |
| 9065632 | 1 | 0.142857143 |  | 9077264 | 1 | 0.2 | |  |  |  |
| 9065973 | 1 | 0.142857143 |  | 9077436 | 1 | 0.2 | |  |  |  |
| 9066259 | 4 | 0.571428571 |  | 9077581 | 3 | 0.6 | |  |  |  |
| 9066874 | 2 | 0.285714286 |  | 9077803 | 1 | 0.2 | |  |  |  |
| 9068530 | 4 | 0.571428571 |  | 9077902 | 1 | 0.2 | |  |  |  |
| 9069689 | 1 | 0.142857143 |  | 9077984 | 1 | 0.2 | |  |  |  |
| 9069792 | 2 | 0.285714286 |  | 9078021 | 1 | 0.2 | |  |  |  |
| 9069892 | 2 | 0.285714286 |  | 9078190 | 1 | 0.2 | |  |  |  |
| 9070079 | 4 | 0.571428571 |  | 9078204 | 1 | 0.2 | |  |  |  |
| 9070194 | 7 | 1 |  | 9078426 | 1 | 0.2 | |  |  |  |
| 9070225 | 7 | 1 |  | 9078545 | 1 | 0.2 | |  |  |  |
| 9070732 | 1 | 0.142857143 |  | 9078567 | 1 | 0.2 | |  |  |  |
| 9070837 | 7 | 1 |  | 9078952 | 5 | 1 | |  |  |  |
| 9071763 | 7 | 1 |  | 9079077 | 1 | 0.2 | |  |  |  |
| 9071834 | 2 | 0.285714286 |  | 9079156 | 1 | 0.2 | |  |  |  |
| 9071924 | 1 | 0.142857143 |  | 9079450 | 1 | 0.2 | |  |  |  |
| 9072221 | 2 | 0.285714286 |  | 9079864 | 1 | 0.2 | |  |  |  |
| 9072313 | 1 | 0.142857143 |  | 9080103 | 3 | 0.6 | |  |  |  |
| 9072742 | 1 | 0.142857143 |  | 9080110 | 3 | 0.6 | |  |  |  |
| 9072875 | 2 | 0.285714286 |  | 9080149 | 1 | 0.2 | |  |  |  |
| 9072975 | 2 | 0.285714286 |  | 9080247 | 1 | 0.2 | |  |  |  |
| 9074073 | 2 | 0.285714286 |  | 9080249 | 1 | 0.2 | |  |  |  |
| 9074112 | 1 | 0.142857143 |  | 9080267 | 1 | 0.2 | |  |  |  |
| 9074950 | 1 | 0.142857143 |  | 9080271 | 4 | 0.8 | |  |  |  |
| 9075021 | 1 | 0.142857143 |  | 9080281 | 5 | 1 | |  |  |  |
| 9075217 | 1 | 0.142857143 |  | 9080289 | 1 | 0.2 | |  |  |  |
| 9075346 | 1 | 0.142857143 |  | 9080297 | 1 | 0.2 | |  |  |  |
| 9075565 | 4 | 0.571428571 |  | 9080301 | 1 | 0.2 | |  |  |  |
| 9075584 | 1 | 0.142857143 |  | 9080343 | 5 | 1 | |  |  |  |
| 9075635 | 1 | 0.142857143 |  | 9080357 | 2 | 0.4 | |  |  |  |
| 9075737 | 5 | 0.714285714 |  | 9080362 | 1 | 0.2 | |  |  |  |
| 9075969 | 1 | 0.142857143 |  | 9080364 | 2 | 0.4 | |  |  |  |
| 9076083 | 1 | 0.142857143 |  | 9080462 | 4 | 0.8 | |  |  |  |
| 9076163 | 1 | 0.142857143 |  | 9081010 | 1 | 0.2 | |  |  |  |
| 9076278 | 1 | 0.142857143 |  | 9081182 | 1 | 0.2 | |  |  |  |
| 9076728 | 1 | 0.142857143 |  | 9081767 | 5 | 1 | |  |  |  |
| 9076858 | 1 | 0.142857143 |  | 9081888 | 1 | 0.2 | |  |  |  |
| 9076929 | 1 | 0.142857143 |  | 9082514 | 4 | 0.8 | |  |  |  |
| 9076950 | 1 | 0.142857143 |  | 9083143 | 2 | 0.4 | |  |  |  |
| 9076991 | 1 | 0.142857143 |  | 9083174 | 2 | 0.4 | |  |  |  |
| 9077060 | 1 | 0.142857143 |  | 9083317 | 1 | 0.2 | |  |  |  |
| 9077196 | 4 | 0.571428571 |  | 9083427 | 1 | 0.2 | |  |  |  |
| 9077264 | 1 | 0.142857143 |  | 9083457 | 2 | 0.4 | |  |  |  |
| 9077436 | 1 | 0.142857143 |  | 9083659 | 1 | 0.2 | |  |  |  |
| 9077581 | 2 | 0.285714286 |  | 9083791 | 1 | 0.2 | |  |  |  |
| 9077803 | 1 | 0.142857143 |  | 9084183 | 3 | 0.6 | |  |  |  |
| 9077902 | 1 | 0.142857143 |  | 9084216 | 2 | 0.4 | |  |  |  |
| 9077984 | 1 | 0.142857143 |  | 9084299 | 3 | 0.6 | |  |  |  |
| 9078021 | 1 | 0.142857143 |  | 9085004 | 1 | 0.2 | |  |  |  |
| 9078190 | 1 | 0.142857143 |  | 9085315 | 1 | 0.2 | |  |  |  |
| 9078204 | 1 | 0.142857143 |  | 9085643 | 1 | 0.2 | |  |  |  |
| 9078426 | 1 | 0.142857143 |  | 9085958 | 1 | 0.2 | |  |  |  |
| 9078545 | 1 | 0.142857143 |  | 9086123 | 2 | 0.4 | |  |  |  |
| 9078567 | 1 | 0.142857143 |  | 9086145 | 1 | 0.2 | |  |  |  |
| 9078832 | 1 | 0.142857143 |  | 9086318 | 2 | 0.4 | |  |  |  |
| 9078888 | 1 | 0.142857143 |  | 9086507 | 1 | 0.2 | |  |  |  |
| 9078952 | 6 | 0.857142857 |  | 9086819 | 1 | 0.2 | |  |  |  |
| 9079077 | 1 | 0.142857143 |  | 9087160 | 1 | 0.2 | |  |  |  |
| 9079156 | 1 | 0.142857143 |  | 9087615 | 5 | 1 | |  |  |  |
| 9079450 | 1 | 0.142857143 |  | 9087758 | 2 | 0.4 | |  |  |  |
| 9079864 | 1 | 0.142857143 |  | 9088017 | 5 | 1 | |  |  |  |
| 9080103 | 3 | 0.428571429 |  | 9088330 | 2 | 0.4 | |  |  |  |
| 9080110 | 5 | 0.714285714 |  | 9088722 | 4 | 0.8 | |  |  |  |
| 9080149 | 1 | 0.142857143 |  | 9088772 | 2 | 0.4 | |  |  |  |
| 9080247 | 1 | 0.142857143 |  | 9090182 | 1 | 0.2 | |  |  |  |
| 9080249 | 1 | 0.142857143 |  | 9090531 | 2 | 0.4 | |  |  |  |
| 9080271 | 3 | 0.428571429 |  | 9090784 | 1 | 0.2 | |  |  |  |
| 9080281 | 4 | 0.571428571 |  | 9092086 | 4 | 0.8 | |  |  |  |
| 9080343 | 6 | 0.857142857 |  | 9092184 | 3 | 0.6 | |  |  |  |
| 9080359 | 1 | 0.142857143 |  | 9092212 | 1 | 0.2 | |  |  |  |
| 9080362 | 4 | 0.571428571 |  | 9092214 | 2 | 0.4 | |  |  |  |
| 9080462 | 7 | 1 |  |  |  |  | |  |  |  |
| 9081182 | 3 | 0.428571429 |  |  |  |  | |  |  |  |
| 9081624 | 1 | 0.142857143 |  |  |  |  | |  |  |  |
| 9081767 | 6 | 0.857142857 |  |  |  |  | |  |  |  |
| 9081888 | 2 | 0.285714286 |  |  |  |  | |  |  |  |
| 9082280 | 1 | 0.142857143 |  |  |  |  | |  |  |  |
| 9082514 | 4 | 0.571428571 |  |  |  |  | |  |  |  |
| 9083124 | 1 | 0.142857143 |  |  |  |  | |  |  |  |
| 9083148 | 1 | 0.142857143 |  |  |  |  | |  |  |  |
| 9083427 | 3 | 0.428571429 |  |  |  |  | |  |  |  |
| 9083457 | 3 | 0.428571429 |  |  |  |  | |  |  |  |
| 9083791 | 2 | 0.285714286 |  |  |  |  | |  |  |  |
| 9084183 | 3 | 0.428571429 |  |  |  |  | |  |  |  |
| 9084299 | 3 | 0.428571429 |  |  |  |  | |  |  |  |
| 9084954 | 1 | 0.142857143 |  |  |  |  | |  |  |  |
| 9085004 | 3 | 0.428571429 |  |  |  |  | |  |  |  |
| 9085643 | 3 | 0.428571429 |  |  |  |  | |  |  |  |
| 9085958 | 2 | 0.285714286 |  |  |  |  | |  |  |  |
| 9086145 | 2 | 0.285714286 |  |  |  |  | |  |  |  |
| 9086318 | 3 | 0.428571429 |  |  |  |  | |  |  |  |
| 9086819 | 3 | 0.428571429 |  |  |  |  | |  |  |  |
| 9087615 | 7 | 1 |  |  |  |  | |  |  |  |
| 9087758 | 3 | 0.428571429 |  |  |  |  | |  |  |  |
| 9088017 | 6 | 0.857142857 |  |  |  |  | |  |  |  |
| 9088330 | 3 | 0.428571429 |  |  |  |  | |  |  |  |
| 9088722 | 4 | 0.571428571 |  |  |  |  | |  |  |  |
| 9088772 | 3 | 0.428571429 |  |  |  |  | |  |  |  |
| 9090182 | 2 | 0.285714286 |  |  |  |  | |  |  |  |
| 9090784 | 2 | 0.285714286 |  |  |  |  | |  |  |  |
| 9091924 | 1 | 0.142857143 |  |  |  |  | |  |  |  |
| 9092086 | 4 | 0.571428571 |  |  |  |  | |  |  |  |
| 9092184 | 2 | 0.285714286 |  |  |  |  | |  |  |  |
| 9092212 | 1 | 0.142857143 |  |  |  |  | |  |  |  |
