# Supplementary material for: MUC16 overexpression induced by gene mutations promotes lung cancer cell growth and invasion
Source: Oncotarget. 2018 Jan 12;9(15):12226–39. doi: 10.18632/oncotarget.24203 (PMC5844741; doi:10.18632/oncotarget.24203)
Supplement: Supplementary file 4 [file oncotarget-09-12226-s004.docx]

| **Supplementary Table 6: The distribution of mutations within the *MUC16* gene.** | | | | |  | | |  |  | |  |
| --- | --- | --- | --- | --- | --- | --- | --- | --- | --- | --- | --- |
| **Sheet 2: The specific mutations within the *MUC16* gene related to the *MUC16* mRNA up-regulation and the *MUC16* mRNA unchanged/down-regulation** | | | | | | | | | |  | |
| **Specific mutations in *MUC16* up-regulated tissue samples** | **Total number** | **Mutation rate** |  | **Specific mutations in *MUC16* down-regulated/unchanged tissue samples** | | **Total number** | **Mutation rate** | |  |  |  |
| 8960007 | 1 | 0.142857143 |  | 8959496 | | 1 | 0.2 | |  |  |  |
| 8961272 | 1 | 0.142857143 |  | 8967182 | | 1 | 0.2 | |  |  |  |
| 8962489 | 1 | 0.142857143 |  | 8969206 | | 1 | 0.2 | |  |  |  |
| 8966181 | 1 | 0.142857143 |  | 8969859 | | 1 | 0.2 | |  |  |  |
| 8967137 | 1 | 0.142857143 |  | 8972240 | | 1 | 0.2 | |  |  |  |
| 8968009 | 1 | 0.142857143 |  | 8974745 | | 1 | 0.2 | |  |  |  |
| 8968981 | 1 | 0.142857143 |  | 8982571 | | 1 | 0.2 | |  |  |  |
| 8969079 | 1 | 0.142857143 |  | 8986085 | | 1 | 0.2 | |  |  |  |
| 8969553 | 1 | 0.142857143 |  | 8993113 | | 1 | 0.2 | |  |  |  |
| 8969715 | 1 | 0.142857143 |  | 8995566 | | 1 | 0.2 | |  |  |  |
| 8969826 | 1 | 0.142857143 |  | 9003615 | | 1 | 0.2 | |  |  |  |
| 8970292 | 1 | 0.142857143 |  | 9004371 | | 1 | 0.2 | |  |  |  |
| 8970357 | 1 | 0.142857143 |  | 9004424 | | 1 | 0.2 | |  |  |  |
| 8970770 | 1 | 0.142857143 |  | 9007089 | | 1 | 0.2 | |  |  |  |
| 8971191 | 1 | 0.142857143 |  | 9007245 | | 1 | 0.2 | |  |  |  |
| 8973772 | 1 | 0.142857143 |  | 9008017 | | 1 | 0.2 | |  |  |  |
| 8974222 | 1 | 0.142857143 |  | 9009851 | | 3 | 0.6 | |  |  |  |
| 8974455 | 1 | 0.142857143 |  | 9015579 | | 1 | 0.2 | |  |  |  |
| 8974733 | 1 | 0.142857143 |  | 9016251 | | 1 | 0.2 | |  |  |  |
| 8975070 | 1 | 0.142857143 |  | 9018301 | | 1 | 0.2 | |  |  |  |
| 8975120 | 1 | 0.142857143 |  | 9019080 | | 1 | 0.2 | |  |  |  |
| 8976016 | 3 | 0.428571429 |  | 9020185 | | 1 | 0.2 | |  |  |  |
| 8976096 | 1 | 0.142857143 |  | 9021885 | | 1 | 0.2 | |  |  |  |
| 8976598 | 1 | 0.142857143 |  | 9022213 | | 1 | 0.2 | |  |  |  |
| 8977313 | 2 | 0.285714286 |  | 9022450 | | 1 | 0.2 | |  |  |  |
| 8978081 | 1 | 0.142857143 |  | 9023732 | | 2 | 0.4 | |  |  |  |
| 8978384 | 1 | 0.142857143 |  | 9024488 | | 1 | 0.2 | |  |  |  |
| 8978409 | 1 | 0.142857143 |  | 9028976 | | 1 | 0.2 | |  |  |  |
| 8980038 | 1 | 0.142857143 |  | 9034358 | | 1 | 0.2 | |  |  |  |
| 8980378 | 1 | 0.142857143 |  | 9034725 | | 1 | 0.2 | |  |  |  |
| 8980604 | 1 | 0.142857143 |  | 9039355 | | 1 | 0.2 | |  |  |  |
| 8982271 | 3 | 0.428571429 |  | 9042826 | | 1 | 0.2 | |  |  |  |
| 8982303 | 1 | 0.142857143 |  | 9042829 | | 2 | 0.4 | |  |  |  |
| 8982497 | 1 | 0.142857143 |  | 9042848 | | 1 | 0.2 | |  |  |  |
| 8984511 | 3 | 0.428571429 |  | 9043142 | | 1 | 0.2 | |  |  |  |
| 8984900 | 1 | 0.142857143 |  | 9043254 | | 1 | 0.2 | |  |  |  |
| 8986327 | 1 | 0.142857143 |  | 9043930 | | 1 | 0.2 | |  |  |  |
| 8986366 | 1 | 0.142857143 |  | 9046883 | | 1 | 0.2 | |  |  |  |
| 8987863 | 1 | 0.142857143 |  | 9048102 | | 1 | 0.2 | |  |  |  |
| 8988850 | 2 | 0.285714286 |  | 9048546 | | 1 | 0.2 | |  |  |  |
| 8989037 | 3 | 0.428571429 |  | 9050570 | | 1 | 0.2 | |  |  |  |
| 8989249 | 1 | 0.142857143 |  | 9051315 | | 1 | 0.2 | |  |  |  |
| 8989582 | 1 | 0.142857143 |  | 9053596 | | 1 | 0.2 | |  |  |  |
| 8990467 | 1 | 0.142857143 |  | 9053616 | | 1 | 0.2 | |  |  |  |
| 8990707 | 1 | 0.142857143 |  | 9054853 | | 1 | 0.2 | |  |  |  |
| 8990912 | 1 | 0.142857143 |  | 9062880 | | 1 | 0.2 | |  |  |  |
| 8991939 | 1 | 0.142857143 |  | 9080267 | | 1 | 0.2 | |  |  |  |
| 8994784 | 5 | 0.714285714 |  | 9080289 | | 1 | 0.2 | |  |  |  |
| 9000129 | 1 | 0.142857143 |  | 9080297 | | 1 | 0.2 | |  |  |  |
| 9003962 | 1 | 0.142857143 |  | 9080301 | | 1 | 0.2 | |  |  |  |
| 9004391 | 1 | 0.142857143 |  | 9080357 | | 2 | 0.4 | |  |  |  |
| 9007873 | 1 | 0.142857143 |  | 9080364 | | 2 | 0.4 | |  |  |  |
| 9008201 | 1 | 0.142857143 |  | 9081010 | | 1 | 0.2 | |  |  |  |
| 9009762 | 1 | 0.142857143 |  | 9083143 | | 2 | 0.4 | |  |  |  |
| 9010222 | 1 | 0.142857143 |  | 9083174 | | 2 | 0.4 | |  |  |  |
| 9010754 | 1 | 0.142857143 |  | 9083317 | | 1 | 0.2 | |  |  |  |
| 9011781 | 1 | 0.142857143 |  | 9083659 | | 1 | 0.2 | |  |  |  |
| 9012612 | 1 | 0.142857143 |  | 9084216 | | 2 | 0.4 | |  |  |  |
| 9013231 | 5 | 0.714285714 |  | 9085315 | | 1 | 0.2 | |  |  |  |
| 9013918 | 1 | 0.142857143 |  | 9086123 | | 2 | 0.4 | |  |  |  |
| 9015049 | 1 | 0.142857143 |  | 9086507 | | 1 | 0.2 | |  |  |  |
| 9015617 | 1 | 0.142857143 |  | 9087160 | | 1 | 0.2 | |  |  |  |
| 9017045 | 1 | 0.142857143 |  | 9090531 | | 2 | 0.4 | |  |  |  |
| 9020549 | 1 | 0.142857143 |  | 9092214 | | 2 | 0.4 | |  |  |  |
| 9021762 | 1 | 0.142857143 |  |  | |  |  | |  |  |  |
| 9025943 | 1 | 0.142857143 |  |  | |  |  | |  |  |  |
| 9026813 | 1 | 0.142857143 |  |  | |  |  | |  |  |  |
| 9030824 | 1 | 0.142857143 |  |  | |  |  | |  |  |  |
| 9037307 | 1 | 0.142857143 |  |  | |  |  | |  |  |  |
| 9037524 | 1 | 0.142857143 |  |  | |  |  | |  |  |  |
| 9042586 | 1 | 0.142857143 |  |  | |  |  | |  |  |  |
| 9042840 | 1 | 0.142857143 |  |  | |  |  | |  |  |  |
| 9042860 | 1 | 0.142857143 |  |  | |  |  | |  |  |  |
| 9045059 | 1 | 0.142857143 |  |  | |  |  | |  |  |  |
| 9045086 | 1 | 0.142857143 |  |  | |  |  | |  |  |  |
| 9045602 | 1 | 0.142857143 |  |  | |  |  | |  |  |  |
| 9046863 | 1 | 0.142857143 |  |  | |  |  | |  |  |  |
| 9051617 | 1 | 0.142857143 |  |  | |  |  | |  |  |  |
| 9053507 | 1 | 0.142857143 |  |  | |  |  | |  |  |  |
| 9056323 | 1 | 0.142857143 |  |  | |  |  | |  |  |  |
| 9057780 | 1 | 0.142857143 |  |  | |  |  | |  |  |  |
| 9058958 | 1 | 0.142857143 |  |  | |  |  | |  |  |  |
| 9064548 | 1 | 0.142857143 |  |  | |  |  | |  |  |  |
| 9065619 | 1 | 0.142857143 |  |  | |  |  | |  |  |  |
| 9065973 | 1 | 0.142857143 |  |  | |  |  | |  |  |  |
| 9069689 | 1 | 0.142857143 |  |  | |  |  | |  |  |  |
| 9070732 | 1 | 0.142857143 |  |  | |  |  | |  |  |  |
| 9074112 | 1 | 0.142857143 |  |  | |  |  | |  |  |  |
| 9078832 | 1 | 0.142857143 |  |  | |  |  | |  |  |  |
| 9078888 | 1 | 0.142857143 |  |  | |  |  | |  |  |  |
| 9080359 | 1 | 0.142857143 |  |  | |  |  | |  |  |  |
| 9081624 | 1 | 0.142857143 |  |  | |  |  | |  |  |  |
| 9082280 | 1 | 0.142857143 |  |  | |  |  | |  |  |  |
| 9083124 | 1 | 0.142857143 |  |  | |  |  | |  |  |  |
| 9083148 | 1 | 0.142857143 |  |  | |  |  | |  |  |  |
| 9084954 | 1 | 0.142857143 |  |  | |  |  | |  |  |  |
| 9091924 | 1 | 0.142857143 |  |  | |  |  | |  |  |  |
